# Ampliconic Genes on the Great Ape Y Chromosomes: Rapid Evolution of Copy Number but Conservation of Expression Levels

**DOI:** 10.1093/gbe/evaa088

**Published:** 2020-05-06

**Authors:** Rahulsimham Vegesna, Marta Tomaszkiewicz, Oliver A Ryder, Rebeca Campos-Sánchez, Paul Medvedev, Michael DeGiorgio, Kateryna D Makova

**Affiliations:** e1 Bioinformatics and Genomics Graduate Program, The Huck Institutes for the Life Sciences, Pennsylvania State University, University Park; e2 Department of Biology, Pennsylvania State University, University Park; e3 Institute for Conservation Research, San Diego Zoo Global, San Diego, California; e4 Department of Biochemistry and Molecular Biology, Pennsylvania State University, University Park; e5 Department of Computer Science and Engineering, Pennsylvania State University, University Park; e6 Center for Computational Biology and Bioinformatics, Pennsylvania State University, University Park; e7 Center for Medical Genomics, Pennsylvania State University, University Park; e8 Institute for Computational and Data Science, Pennsylvania State University, University Park

**Keywords:** great apes, Y chromosome, ampliconic genes, gene copy number, gene expression, bonobo, orangutan

## Abstract

Multicopy ampliconic gene families on the Y chromosome play an important role in spermatogenesis. Thus, studying their genetic variation in endangered great ape species is critical. We estimated the sizes (copy number) of nine Y ampliconic gene families in population samples of chimpanzee, bonobo, and orangutan with droplet digital polymerase chain reaction, combined these estimates with published data for human and gorilla, and produced genome-wide testis gene expression data for great apes. Analyzing this comprehensive data set within an evolutionary framework, we, first, found high inter- and intraspecific variation in gene family size, with larger families exhibiting higher variation as compared with smaller families, a pattern consistent with random genetic drift. Second, for four gene families, we observed significant interspecific size differences, sometimes even between sister species—chimpanzee and bonobo. Third, despite substantial variation in copy number, Y ampliconic gene families’ expression levels did not differ significantly among species, suggesting dosage regulation. Fourth, for three gene families, size was positively correlated with gene expression levels across species, suggesting that, given sufficient evolutionary time, copy number influences gene expression. Our results indicate high variability in size but conservation in gene expression levels in Y ampliconic gene families, significantly advancing our understanding of Y-chromosome evolution in great apes.

## Introduction

Great apes (family Hominidae) include four genera—*Pongo* (Bornean, Sumatran, and Tapanuli orangutans), *Gorilla* (eastern and western gorillas), *Pan* (common chimpanzee and bonobo), and *Homo* (humans)—who shared a common ancestor ∼13 Ma (Glazko and Nei 2003). All great apes but humans are endangered species (Ancrenaz et al. 2016; Fruth et al. 2016; Humle et al. 2016; Maisels et al. 2016; Singleton et al. 2017). Therefore, understanding genetic variation within and among species, and preserving reproductive fitness of these animals, is of utmost importance. Some of the clues to addressing these pressing questions lie in the analysis of sex chromosomes of great apes. Sex chromosomes harbor genes linked to spermatogenesis and that influence fertility and reproduction (Skaletsky et al. 2003; Ross et al. 2005); however, we lack a key understanding of the diversity of these genes across great apes.

The great ape sex chromosomes, X and Y, originated from a pair of homologous chromosomes in the common ancestor of therian mammals 160–190 Ma (Veyrunes et al. 2008; Luo et al. 2011). Over time, the X chromosome has retained most of its ancestral gene content facilitated by continued recombination in females (Mueller et al. 2013), whereas the Y chromosome underwent a series of inversions and lost most of its genes due to the lack of recombination with the X (Charlesworth D and Charlesworth B 1980; Skaletsky et al. 2003). Additionally, sexual antagonism led to accumulation of genes and mutations beneficial to males on the Y (Fisher 1931). Since the split of great apes from their common ancestor, the Y chromosome continued to diverge. Cytogenetic studies have demonstrated that the Y chromosome differs in size, gene content, and gene order among great ape species (Gläser et al. 1998). To date, among great apes, only the human, chimpanzee, and gorilla Y chromosomes have been sequenced and assembled, with the gorilla assembly being in draft state (Skaletsky et al. 2003; Hughes et al. 2010; Tomaszkiewicz et al. 2016). The Y chromosomes of other great ape species are yet to be deciphered.

The same sequence regions are present on the Y chromosomes of great apes studied to date. These include the pseudoautosomal region and the male-specific region (MSY, or the male-specific region on the Y) (Skaletsky et al. 2003; Hughes et al. 2012). The pseudoautosomal region can recombine with the X and thus is identical to the homologous region on it (Lahn and Page 1999; Graves 1995). The MSY region in great apes is interspersed with heterochromatic (Cechova et al. 2019) and euchromatic sequences of different sizes. The euchromatic MSY portion consists of single-copy X-degenerate and X-transposed regions and of highly repetitive ampliconic regions (Skaletsky et al. 2003; Hughes et al. 2010, 2012; Tomaszkiewicz et al. 2016). The X-degenerate regions constitute the remnants of the ancient proto-sex chromosomes, whereas the X-transposed region (so far found only in human) represents a recent transposition from the X to the Y. The ampliconic regions harbor protein-coding multicopy gene families that are expressed in testis and are associated with spermatogenesis and male fertility (Rozen et al. 2003; Skaletsky et al. 2003; Hughes et al. 2010, 2012; Tomaszkiewicz et al. 2016). In humans, these are *BPY2* (basic protein Y2), *CDY* (chromodomain Y), *DAZ* (deleted in azoospermia), *HSFY* (heat-shock transcription factor Y), *PRY* (PTP-BL related Y), *RBMY* (RNA-binding motif Y), *TSPY* (testis-specific Y), *VCY* (variable charge), and *XKRY* (X Kell blood-related Y) (Skaletsky et al. 2003). Five of these nine gene families—*BPY2*, *CDY*, *DAZ*, *RBMY*, and *TSPY*—are shared among great apes studied so far, however, information about presence/absence of the other four gene families in different great ape species remains incomplete (reviewed by Hallast and Jobling [2017]). The majority of ampliconic gene families in human and chimpanzee are located in palindromes—large inverted repeats common on the Y chromosome (Rozen et al. 2003; Skaletsky et al. 2003; Hughes et al. 2010). The exception to this pattern is the *TSPY* gene family, which in humans is present as a tandem array outside palindromes (Skaletsky et al. 2003). The presence of palindromes facilitates gene conversion between ampliconic genes, which can remove deleterious mutations and lower sequence diversity within Y ampliconic gene families (Rozen et al. 2003; Hallast et al. 2013). Additionally, ampliconic sequences facilitate gene conversion and allow for the decoupling between deleterious and beneficial mutations speeding up adaptation (Connallon and Clark 2010; Marais et al. 2010; Betrán et al. 2012; Trombetta and Cruciani 2017). A comprehensive investigation of the evolutionary dynamics of copy number and expression of Y ampliconic gene families has been lacking to date.

Several studies indicated intraspecific variation in Y-chromosome ampliconic gene copy number in great apes (Repping et al. 2006; Hughes et al. 2010; Schaller et al. 2010; Oetjens et al. 2016; Tomaszkiewicz et al. 2016; Skov et al. 2017; Lucotte et al. 2018; Ye et al. 2018). High variation in gene copy number for the *RBMY* and *TSPY* gene families was observed in humans, chimpanzees, and gorillas (Oetjens et al. 2016; Tomaszkiewicz et al. 2016; Skov et al. 2017; Lucotte et al. 2018; Ye et al. 2018; Vegesna et al. 2019). Chimpanzees also exhibit high-copy-number variation in the *DAZ* gene family (Schaller et al. 2010; Oetjens et al. 2016) and gorillas—in the *CDY* and *HSFY* gene families (Tomaszkiewicz et al. 2016). Targeted fluorescence in situ hybridization intraspecific analysis of the *DAZ* and *CDY* gene families identified no variation in Bornean orangutan but two variants in Sumatran orangutan (Greve et al. 2011). Thus, the precise range of copy number variation for Y ampliconic gene families remains unknown in either of these two orangutan species. The available information on copy number variation for Y ampliconic gene families in bonobos is currently limited to two individuals (Oetjens et al. 2016). Thus, we are critically missing data on ampliconic gene copy number variation in orangutans and bonobos. Moreover, variation in Y ampliconic gene copy number in great apes has never been analyzed in an evolutionary framework.

The evolution of gene expression of Y ampliconic gene families in great apes has remained even more understudied. Recently, we demonstrated dosage regulation of human Y ampliconic gene expression in testis when compared with their homologs on the X or autosomes (Vegesna et al. 2019). Additionally, expression levels and Y haplogroup or gene copy number of an individual were not significantly associated with each other (Vegesna et al. 2019). However, across gene families, we observed a positive correlation between the copy number and expression levels (Vegesna et al. 2019), which was also shown in another study examining expression of Y ampliconic gene families at different stages of spermatogenesis (Lucotte et al. 2018). Apart from these few studies, little is known about variation and evolution in expression of Y ampliconic gene families in great apes. Moreover, the relationship between copy number and expression levels for the Y ampliconic genes in nonhuman great ape species remains unexplored.

Previous studies suggested that evolution of the Y chromosome could reflect different mating patterns and social structure in great apes (Hughes et al. 2010; Schaller et al. 2010; Hallast et al. 2016). Great apes exhibit substantial variation in mating systems, which can result in different levels of sperm competition. Bonobos and chimpanzees have a multimale–multifemale, that is, polygynandrous, mating system, where female promiscuity results in high levels of sperm competition. In contrast, gorillas have a unimale–multifemale, that is, polygynous, mating system, which results in low levels of sperm competition. Orangutans and humans fall in between (Wistuba et al. 2003; Harrison and Chivers 2007). The roving male polygynous mating system in orangutans, and mating systems defined from monogamous to polygynous in humans, result in levels of sperm competition that are lower than those in chimpanzees/bonobos and higher than those in gorillas (Wistuba et al. 2003; Harrison and Chivers 2007). Based on the importance of Y ampliconic gene families in spermatogenesis, it is reasonable to hypothesize that their copy number and/or expression levels can be different among great apes with various mating patterns and different levels of sperm competition and can be associated with sperm phenotypes.

In this study, we present the first comprehensive analysis of the evolution of Y ampliconic gene copy number and expression levels across great apes. Using droplet digital polymerase chain reaction (ddPCR), we estimated copy number of ampliconic gene families in nonhuman great apes. We tested whether the copy number of Y ampliconic gene families is conserved across great apes and identified species that have experienced a significant gain or loss in copy number. Additionally, we generated testis expression data for bonobo and Bornean orangutan, thus augmenting such data we and others previously generated for gorilla, orangutan, chimpanzee, and human (Brawand et al. 2011; Ardlie et al. 2015; Ruiz-Orera et al. 2015; Carelli et al. 2016; Fungtammasan et al. 2016; Tomaszkiewicz et al. 2016). We assembled the transcripts of Y ampliconic gene families and tested whether their expression is conserved across great apes. Next, we investigated the evolutionary relationship between the Y ampliconic gene families’ copy number and expression. Our results highlight the important role of ampliconic genes in shaping Y-chromosome evolution and evolution of great apes in general.

## Materials and Methods

### DNA Samples

DNA samples (supplementary table S1, Supplementary Material online) from seven bonobos, six Bornean orangutans, and four Sumatran orangutans, as well as a blood sample from an additional Sumatran orangutan (KB5565) were provided by the San Diego Zoological Society. We extracted DNA from the latter two samples using the DNeasy Blood and Tissue Kit (Qiagen). DNA samples from nine western chimpanzees were provided by Mark Shriver at Pennsylvania State University.

### ddPCR Assays for Ampliconic Gene Copy Number Estimation in Bonobo and Orangutan

The PCR protocol and primers for EvaGreen-based ddPCR assays were designed according to the parameters specified in Tomaszkiewicz et al. (2016), using great ape species-specific sequences of a two-copy *RPP30* and a single-copy *SRY* as references. BWA-MEM alignments (version 0.7.10) (Li 2013) of raw Illumina reads from several male orangutan and bonobo data sets (RNA-Seq data sets present in supplementary table S2, Supplementary Material online, whole-genome-sequence data sets listed below) to the reference gene sequences were visualized in Integrative Genomics Viewer (version 2.3.72) (Thorvaldsdóttir et al. 2013) and consensus sequences were retrieved in order to design the primers. The primers for evaluating the copy number of *BPY2*, *CDY*, *DAZ*, *HSFY*, *PRY*, *RBMY*, and *TSPY* gene families in Bornean and Sumatran orangutans were designed in the protein-coding regions using the gene sequences previously published for Sumatran orangutan (Cortez et al. 2014) and RNA-Seq data sets generated in-house for the Bornean orangutan (supplementary data set S1, Supplementary Material online, and see below). Primers for orangutan *XKRY* (supplementary data set S1, Supplementary Material online) were designed using the published Sumatran orangutan whole-genome sequencing data set (SRR10393305, without gene annotation) and their male-specific presence in genomic DNA of both orangutan species was confirmed by PCR. Primers for *SRY* were designed using a previously published Sumatran orangutan gene sequence (Cortez et al. 2014), and primers for *RPP30* were designed using the Sumatran orangutan reference female genome (ponAbe3). To estimate the copy number of the *BPY2*, *CDY*, *DAZ*, *RBMY*, and *TSPY* gene families in bonobo and chimpanzee, we used previously published primers for gorilla and human (Tomaszkiewicz et al. 2016) that identically matched the chimpanzee Y-specific sequence, and the publicly available whole-genome male bonobo data set (SRR740905). The *VCY* and *SRY* primers for chimpanzees were designed using the chimpanzee Y-chromosome reference sequence (Hughes et al. 2010). The *SRY* primers for bonobo were designed using the whole-genome male bonobo data set generated in-house (supplementary data set S1, Supplementary Material online). The primers for *RPP30* were designed using the chimpanzee (panTro6) and bonobo (panpan1.1) reference female genomes (supplementary data set S1, Supplementary Material online). To complete our copy number data set, we also used the previously published (by our group) copy number data from 14 wild-born gorillas (Tomaszkiewicz et al. 2016) and ten humans with African Y haplogroup (E) (Ye et al. 2018). Each sample was run in at least three replicates (supplementary data set S2, Supplementary Material online) and the mean value was calculated across the replicates (supplementary table S3, Supplementary Material online).

### Construction of Y-Specific Great Ape Phylogenetic Trees

Hallast et al. identified 54,611 positions with intra- and interspecific single-nucleotide variants by comparing a 750,616-bp region across the Y chromosomes of great apes (Hallast et al. 2016). From this data set, we picked sequences of one individual per species (uniformly at random) and generated a distance matrix (pairwise nucleotide differences) using MEGA7 (Kumar et al. 2016). Using the mutation rate on the Y chromosome of humans (8.88 × 10^−10^ mutations per position per year [Helgason et al. 2015]) as a proxy for all great apes, and the number of nucleotide differences among species obtained from the distance matrix above, we estimated the time in years since the most recent common ancestor (TMRCA), where time is defined as (Walsh 2001)

(1) $$\mathrm{TMRCA}\;=\frac{1\;}{2(\mathrm{mutation}\;\mathrm{rate})}\times \log_{e}\left(\frac{\mathrm{length}\;\mathrm{of}\;\mathrm{sequences}}{\mathrm{no}.\;\;\mathrm{of}\;\mathrm{matches}}\right).$$

MEGA7 was used to estimate an unrooted maximum likelihood tree from the same set of sequences employed to compute TMRCA. We generated two separate trees, the first one including all great ape species analyzed (bonobo, chimpanzee, human, gorilla, Sumatran orangutan, and Bornean orangutan) and the second one excluding Sumatran orangutan. The second tree was used in gene expression analysis, in which we lacked expression data from multiple Sumatran orangutan individuals. Both trees were converted to rooted trees using *reroot()* function from ape package in R (Paradis et al. 2004). As a final step, we recalibrated the trees to represent the branch lengths as TMRCA (in thousands of years) using the *chronos()* function in the *ape* package (Paradis et al. 2004). We used the *makeChronosCalib()* function to set the TMCRA for each common ancestor node of great apes and passed it as a parameter to the *chronos()* function, which enforces a molecular clock. Finally, we encoded the trees in Newick format for downstream analysis. The two Newick-formatted trees were ((((Bonobo:2432, Chimp:2432):5641, Human:8073):4633, Gorilla:12706):15346.37,(Borangutan:578, Sorangutan:578):27474.37) and ((((Bonobo:2432, Chimp:2432):5641, Human:8073):4633, Gorilla:12706):15351.43, Borangutan:28057.43).

### Analysis of Conservation in Copy Number across Great Ape Species

We used CAFE[v4.2] (Computational Analysis of gene Family Evolution) (Han et al. 2013) to study the evolution of ampliconic gene family size. CAFE uses a birth-and-death stochastic process to model gene gain or loss along each lineage of a given phylogenetic tree (De Bie et al. 2006). For each of the nine ampliconic gene families, the median gene copy number in each great ape species (supplementary table S4, Supplementary Material online) and the first phylogenetic tree from the previous section were provided as input to CAFE. Using maximum likelihood, CAFE estimated the rate parameter *λ* (rate of gene birth and death) and gene copy numbers of each gene family at the internal nodes of the phylogenetic tree. Based on these maximum likelihood estimates, CAFE computed gene-family-specific *P* values for tests of significant gain or loss of copy number in individual gene families in any particular extant and ancestral great ape species. For each gene family with a significant gene-family-specific *P* value (significance cutoff of 0.05 was used), CAFE also provided a *P* value for every branch on the phylogenetic tree, which indicates the significance of the shift in gene family copy number along the branch. Based on these branch-specific *P* values, we identified gene families that have undergone expansions or contractions along branches on the phylogenetic tree (Bonferroni-corrected *P* value cutoff of 0.05/10 = 0.005; ten nodes in great ape phylogenetic tree).

### RNA-Seq Data Sets

Testis-specific RNA-Seq data sets were obtained for human, bonobo, chimpanzee, gorilla, Bornean orangutan, and Sumatran orangutan (supplementary table S2, Supplementary Material online). The data sets were either publicly available (Brawand et al. 2011; Ardlie et al. 2015; Ruiz-Orera et al. 2015; Carelli et al. 2016; Fungtammasan et al. 2016; Tomaszkiewicz et al. 2016) or generated in-house. The public RNA-Seq data sets included sequences of strand-specific, paired-end libraries (SRR2040590 and SRR2040591 for chimpanzee, SRR2176206 and SRR2176207 for Bornean orangutan, SRR10393299–SRR10393304 for Sumatran orangutan, SRR3053573 and SRR10393358 for gorilla, and SRR1090722 and SRR1077753 for human) and of unstranded libraries (SRR306837 for bonobo, SRR306825 for chimpanzee, and SRR306810 for gorilla) (Brawand et al. 2011; Ardlie et al. 2015; Ruiz-Orera et al. 2015; Carelli et al. 2016). Testis-specific expression data for three humans with African Y-chromosome haplogroup E (SRR817512, SRR1100440, and SRR1102852) were retrieved from the GTEx project (Ardlie et al. 2015). An additional bonobo RNA-Seq library was generated from total RNA extracted from the bonobo whole-testis sample (individual ID 5013, from San Diego Zoological Society) with the RNeasy Mini Kit (Qiagen) and subsequently treated with DNase I (Ambion). Ribosomal RNA was depleted with the RiboZero Gold rRNA removal kit (Epicentre). The cDNA library was generated with the RNA ScriptSeq v2 RNA-Seq library preparation kit (Epicentre) and quantified with Qubit (Life Technologies) and Bioanalyzer (Agilent 2100). RNA sequencing was carried out on MiSeq using 151-bp paired-end sequencing protocol (∼100 million reads were generated). Raw sequence data were deposited in the NCBI Sequence Read Archive under accession numbers SRR10392519–SRR10392521. An additional Bornean orangutan RNA-Seq data set was generated as follows. RNA was extracted from the whole testis from a sample provided by San Diego Zoological Society (individual ID 3405). As in Brawand et al. (2011), Ardlie et al. (2015), Ruiz-Orera et al. (2015), Carelli et al. (2016), Fungtammasan et al. (2016), and Tomaszkiewicz et al. (2016), RNA was extracted, integrity verified with Bioanalyzer, and sequenced on HiSeq2500 after preparing the libraries with TruSeq RNA Sample Prep kit (Illumina). Raw sequence data were deposited in the NCBI Sequence Read Archive under accession numbers SRR10392513–SRR10392518.

Female liver RNA-Seq data sets were obtained from publicly available data sets (SRR306835 for bonobo, SRR306823 for chimpanzee, SRR306808 for gorilla, SRR306798 for orangutan, and SRR1071668 for human) (Brawand et al. 2011; Ardlie et al. 2015; Carithers et al. 2015). They were used to filter out female transcripts during transcriptome assembly.

### Transcriptome Assembly of Y Ampliconic Genes in Great Apes

The reference genomes for great apes—gorGor5 (Gordon et al. 2016), panPan1 (Prüfer et al. 2012), panTro5 (Chimpanzee Sequencing and Analysis Consortium 2005), PonAbe2 (Locke et al. 2011), and hg38—were downloaded from the UCSC Genome Browser (Kent et al. 2002). The transcriptome assembly pipeline was adapted from Tomaszkiewicz et al. (2016). The RNA-Seq reads (from the previous section) were first checked for the presence of TruSeq adapters and then we removed the adapters and low-quality regions using Trimmomatic[v0.36] (Bolger et al. 2014). For each great ape species, its testis RNA-Seq reads were first mapped to their respective female reference genome (reference genome excluding the Y chromosome in case of human and chimpanzee) with Tophat2[v2.1.1] (Kim et al. 2013), and the unmapped reads (enriched for male-specific transcripts) were assembled with Trinity[v2.4.0] (Grabherr et al. 2011; Haas et al. 2013) and SOAPdenovo-Trans[v1.03] (Xie et al. 2014) with k-mer size of 25 bp. Other parameters were set based on read length and insert size required for each particular RNA-Seq data set. The resulting contigs were aligned to the respective female reference genomes with BLAT[v36x2] (Kent 2002), and contigs that aligned at >90% of their length with 100% identity were filtered from subsequent steps. Next, we aligned female liver RNA-Seq reads to the filtered contigs using Bowtie[v1.1.2] (Langmead 2010) and removed contigs that were covered at over 90% of their length by mapped female liver RNA-Seq reads. We combined the contigs from both the Tophat2 and Trinity assemblers and used CD-HIT[v4.7] (Li and Godzik 2006; Fu et al. 2012) to remove redundant sequences. We next scaffolded the remaining contigs using SSPACE[v3.0] (Boetzer et al. 2011). We further mapped testis and female liver RNA-Seq reads to the gene scaffolds with Bowtie[v1.1.2] (Langmead 2010) and retained only male-specific gene scaffolds (with at least 80% of the sequence covered by male-specific reads and no more than 20% of the sequence covered by female-specific reads). From the filtered male-specific scaffolds, we generated consensus sequences using minimus2[v3.1.0] from the AMOS consortium (Sommer et al. 2007). Annotation of the final transcripts was performed using nucleotide and protein databases using BLAST[v2.6.0+] (Altschul et al. 1990). The above pipeline (supplementary fig. S1, Supplementary Material online) was run for each great ape species separately, and the longest transcript representing ampliconic gene family was obtained for each species (supplementary data set S3, Supplementary Material online). The transcripts for genes with low expression were not assembled due to lack of reads covering these genes. We did not set a manual cutoff for low expression, instead we made an assumption that for the genes for which we were unable to assemble the transcripts, the expression was low, as not enough reads were represented in the sample to assemble the transcript.

### Estimating Gene Expression Levels from RNA-Seq Data Sets

To obtain gene expression levels of Y ampliconic gene families, we used the human RefSeq database downloaded from the UCSC Genome Browser (http://hgdownload.soe.ucsc.edu/goldenPath/hg38/bigZips/, last accessed October 2016) as a reference, along with the longest ampliconic gene transcript assembled for the available gene families (see previous section). We generated an index for the reference using the salmon[v0.14.1] index function (Patro et al. 2017) with k-mer size 31 (-k 31 –keepDuplicates). Standard pipelines such as Tophat2 (Kim et al. 2013) and RSEM (Li and Dewey 2011) optimized to align reads to the same species reference could not be used in our case, and so we developed a new pipeline. For each sample, using the salmon *quant* (-l A -p 8 –validateMappings) function, we obtained the read counts per transcript on the available testis samples for each species. The transcript-level read counts were converted to gene level using the tximport package[v1.2.0] (Soneson et al. 2015). The gene-level read counts for the RNA-Seq samples were normalized using DESeq2[v1.14.1] (Love et al. 2014). The testis RNA-Seq data are a mix of paired-end (with technical replicates) and single-end (without replicates) data sets. We observe batch effects at two different levels, when we obtained the normalized read counts for each sample using salmon + DESeq2 pipeline and visualized the relationship among samples using principal component analysis (PCA) plots and heatmap of Euclidean distances between samples as suggested in the DESeq2 workflow (we employed the *rlog()* function in DESeq2 to normalize read counts and then used the *plotPCA()* function from the same package to generate PCA plots and *dist()* to calculate the Euclidean distance between samples): 1) Samples with paired-end reads were clustered together. To overcome this, we processed the paired-end data sets as single-end data. 2) Samples with replicates clustered together (when we collapsed replicates into a single sample using the *collapseReplicates()* function in DESeq2). To overcome this effect, we picked one replicate uniformly at random per sample when available. By simplifying the data set via assuming all samples as single-end data without replicates, we were able to overcome batch effects (supplementary figs. S2–S4, Supplementary Material online). The final PCA plots (supplementary figs. S3 and S4, Supplementary Material online) show clustering of samples by species, which we assumed as biological signal and absence of batch effects.

### Testing for Conservation in Gene Expression Levels

To test whether the expression levels for Y ampliconic gene families are conserved across great ape species, we used the expression variance and evolution (EVE) model (Rohlfs et al. 2014). This model parameterizes the ratio of population to evolutionary expression variance (*β*) taking phylogeny into account, that is, it provides the implementation for phylogenetic analysis of variance (ANOVA). Similar to the *F* statistic (a measure of the ratio of variation between groups to variation within groups) in ANOVA, the *β* parameter estimated by the EVE model represents the ratio of within-species expression variation, to phylogenetically corrected between-species expression variation. The EVE model is based on an Ornstein–Uhlenbeck process (Rohlfs and Nielsen 2015), which models a random walk with a pull toward an optimal value. In the Ornstein–Uhlenbeck process employed by the EVE model, genetic drift (*σ*^2^) is explained by the random walk, the strength of selection (*ɑ*) by the directional pull, and optimal gene expression (*θ*) at the species level by optimal value. The EVE model also has a parameter that captures the variation of expression within species (↓) to estimate *β*. Given the Y-chromosome-specific phylogenetic tree (the second tree without Bornean orangutan was used; see the Construction of Y-specific great ape phylogenetic trees section) and the expression values for the five Y ampliconic gene families found in all great apes from multiple samples per species, the EVE model estimated the above-mentioned parameters and used them to calculate the *β* parameter for each gene family *i* (*β_i_*) and all the gene families together (*β*_shared)_. The EVE model was then used to test whether the ratio *β_i_* for each gene *i* was similar to all the genes evolving neutrally in the phylogeny (i.e., *β_i_* = *β*_shared_ where *i* indexes each gene in the data set). Deviations from this expectation are suggestive of selection. We tested whether the *β_i_* parameter for any one gene family deviates from this expectation (i.e., *β_i_* ≠ *β*_shared_). If *β_i_* > *β*_shared_, then there is more variation within species than between species at gene *i* compared with expected, which could be suggestive of diversifying selection within species. Conversely, if *β_i_* < *β*_shared_, then there is more variation between species than within species at gene *i* compared with expected, which could be indicative of directional selection along extant or ancestral branches of the phylogeny. We used the -S parameter in the EVE model to perform the expression divergence/diversity test on each of the five gene families (-n 5) separately, using the ampliconic gene expression values from the previous section and the Y-chromosome-specific phylogenetic tree as inputs. The EVE model calculated the likelihood ratio between the null and alternative hypotheses (H_o_: *β_i_* = *β*_shared_ vs. H_a_: *β_i_* ≠ *β*_shared_). The likelihood ratios follow a chi-square distribution with one degree of freedom, which makes it possible to convert the likelihood ratios to *P* values. The *P* values are used to infer whether expression levels of gene families tested are conserved across great ape species after taking their phylogenetic relationship into account.

## Results

### Dynamic Evolution of Y Ampliconic Gene Copy Number

#### Estimating Copy Number

To evaluate the copy number of Y ampliconic genes, we used a ddPCR protocol similar to the one utilized in previous studies from our group (Tomaszkiewicz et al. 2016; Ye et al. 2018) (see Materials and Methods). With ddPCR, template DNA is fractionated into multiple droplets within which PCR takes place, and each droplet is analyzed to determine copy number in a sample (Hindson et al. 2011). ddPCR differs from quantitative real-time PCR in that it estimates the absolute quantity of DNA without generating a standard curve (Hindson et al. 2011). It serves as a more economic alternative to whole-genome sequencing for copy number evaluation for targeted genomic regions or gene families, while providing similar copy number estimates (Vegesna et al. 2019), and is particularly attractive in the absence of the Y-chromosome reference (which is the case for bonobo and Sumatran and Bornean orangutans).

**Fig. 1.**
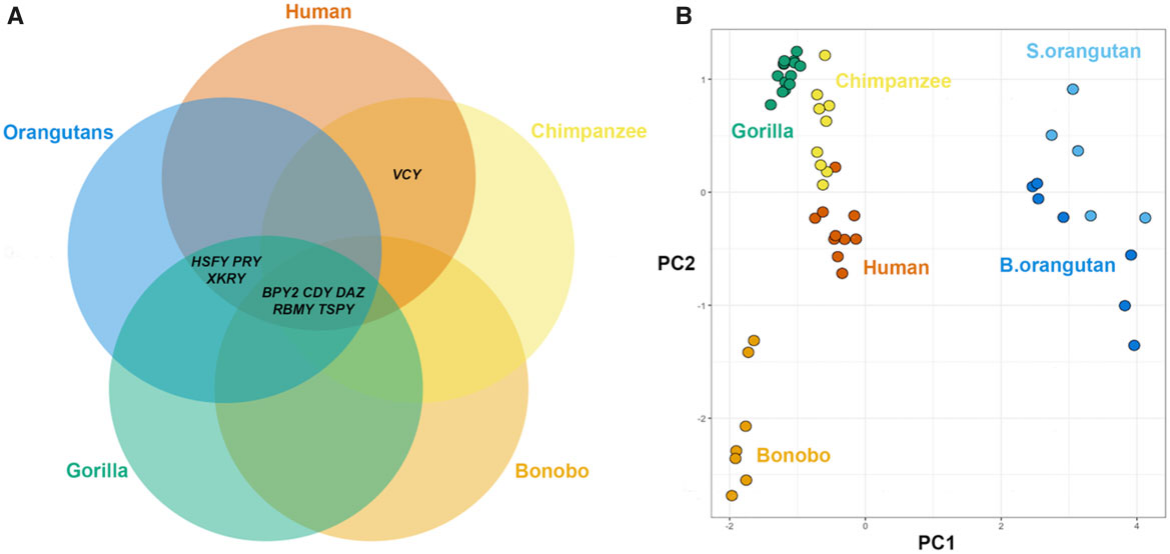
—Y ampliconic genes in great apes. (*A*) Venn diagram showing gene content comparison among great ape species. (*B*) Plot of the first two principal components (PCs) of Y ampliconic gene copy numbers across great ape species (the first and second PCs explained 68.7% and 22.8% of the variation, respectively; supplementary figure S5, Supplementary Material online, shows variation explained by the other components).

Our samples included seven bonobos, nine chimpanzees, seven Bornean orangutans, and five Sumatran orangutans. To the best of our knowledge, all samples came from wild-born, unrelated individuals. Additionally, we used Y ampliconic gene copy number estimates generated by our group previously for 10 humans with African ancestry (Ye et al. 2018) and 14 wild-born gorillas (Tomaszkiewicz et al. 2016). Summarizing the data generated in our study and previous findings (reviewed by Hallast and Jobling [2017]), we show that eight ampliconic gene families—*BPY2*, *CDY*, *DAZ*, *HSFY*, *PRY*, *RBMY*, *TSPY*, and *XKRY*—are shared exclusively among human, gorilla, and both species of orangutan (fig. 1*A*). We demonstrate for the first time that the *XKRY* gene family is present in both Bornean and Sumatran orangutans (fig. 1*A*). *HSFY*, *PRY*, and *XKRY* are pseudogenized in chimpanzee and bonobo, and *VCY* is absent in bonobo, gorilla, and both species of orangutan examined (fig. 1*A*). For these gene families, we assigned a gene count of zero in our analysis. Based on the overall number of Y ampliconic gene copies (with all families combined), individuals can be separated into clusters with PCA (fig. 1*B* and supplementary fig. S5, Supplementary Material online).

#### Copy Number and Its Variance in Individual Gene Families

**Fig. 2.**
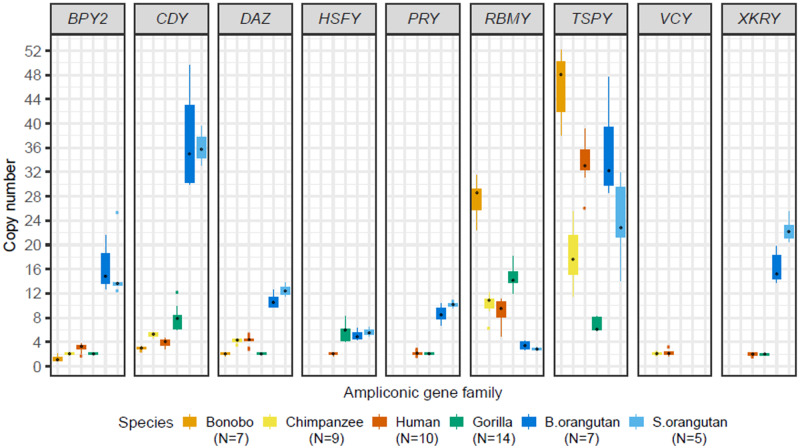
—Variation in copy number of Y ampliconic gene families in great apes. Box plots summarizing the distribution of copy numbers of the six great ape species across nine Y ampliconic gene families. The gene families are separated into individual plots with the gene family name at the top. Within each plot, the *x* axis represents six species (bonobo, chimpanzee, human, gorilla, Bornean orangutan, and Sumatran orangutan) and the *y* axis represents copy number. The black dot within each boxplot is the median value per species.

**Fig. 3.**
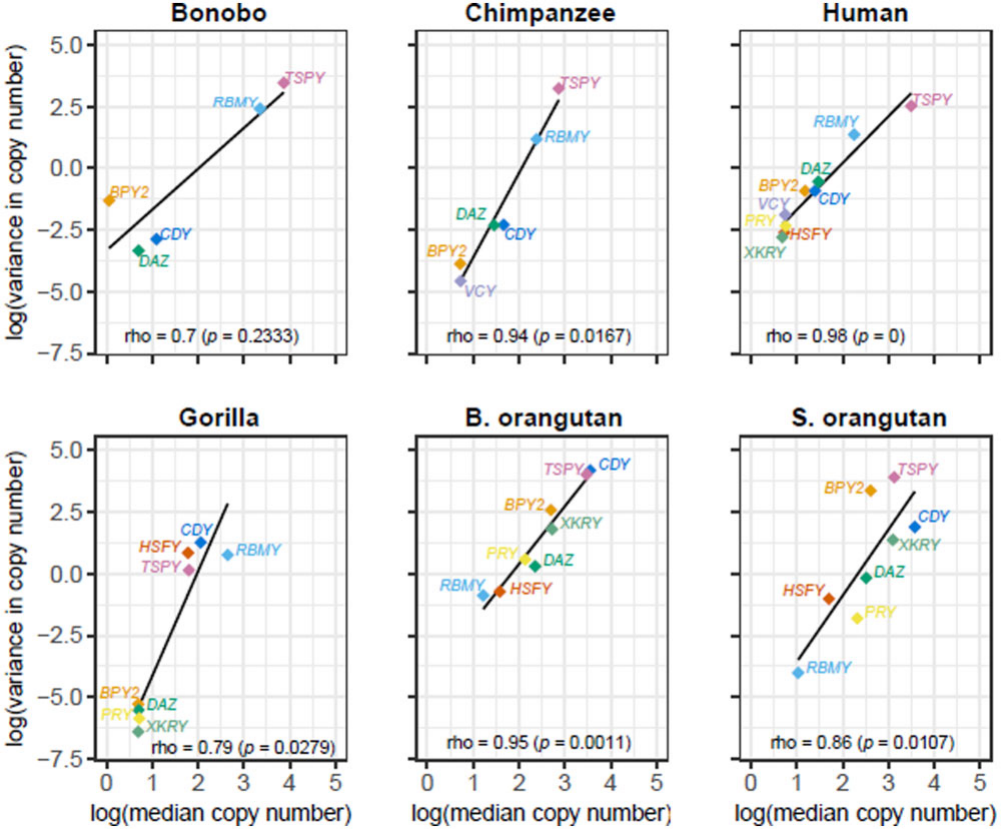
—Larger Y ampliconic gene families are more variable across great apes. The six scatter plots represent the relationship between median copy number and variance for each of the species, and the species name is present at the top of each plot. The *x* axis represents natural logarithm of median copy number and the *y* axis is a natural logarithm of variance in copy number. The Spearman correlations were calculated using the *cor.test()* function in R and the *P* values are in parentheses. The black line represents the linear function fitted to the given data points. The dots are color coded to represent the nine gene families, with missing dots indicating gene family absence in that species.

Separating the data by Y ampliconic gene family and species (fig. 2), we observed a positive correlation between gene family copy number and its variance in each species (fig. 3) and in each gene family (supplementary fig. S6, Supplementary Material online). The *TSPY* gene family had consistently higher copy number and variance than other Y-chromosome ampliconic gene families in bonobo, chimpanzee, and Sumatran orangutan and had the second highest (after *CDY*) copy number and variance in Bornean orangutan (fig. 3). The *RBMY* gene family also had high copy number and variance in all great ape species except for the two orangutan species. In contrast, the *VCY* family, present only in human and chimpanzee, had consistently low copy numbers (figs. 2 and 3).

Most ampliconic gene families were more copious in orangutans than in other species (figs. 2 and 3). For example, the *PRY* and *XKRY* gene families each had at least eight copies in orangutans (Bornean orangutan: 8 copies for *PRY* and 15 copies for *XKRY*; Sumatran orangutan: 10 copies for *PRY* and 22 copies for *XKRY*)—in contrast, in Homininae these gene families were either lost (in bonobo and chimpanzee) or had a median size of only two copies (in human and gorilla). Also, each of the *BPY2*, *CDY*, and *DAZ* gene families had more than twice the number of gene copies in orangutans than that found in other great ape species.

Gene families lost in some species (*HSFY*, *PRY*, *VCY*, and *XKRY*) usually had few copies and low variation in the closely related species. For instance, the *HSFY*, *PRY*, and *XKRY* gene families were pseudogenized in bonobo and chimpanzee, and human had a low copy number (on average two copies) for these gene families (figs. 2 and 3). Similarly, the *VCY* gene family was lost in the majority of great ape species, except for chimpanzee and human, in which it had a low copy number (on average two copies; figs. 2 and 3).

#### Copy Number Differences between Recently Diverged Species

As an initial investigation into how quickly Y ampliconic gene families evolve, we tested whether copy numbers for individual gene families differed significantly between recently diverged, sister species. Two pairs of closely related species were included in this comparison—chimpanzee and bonobo, separated ∼0.77–1.8 Ma (Yu et al. 2003; Hey 2010), and Sumatran and Bornean orangutans, separated ∼0.4 Ma (Locke et al. 2011). Five gene families were tested in bonobo versus chimpanzee, and eight—in Sumatran versus Bornean orangutans (fig. 1*A*), with a permutation test in which we compared the mean copy number difference between the two species (permuting species labels with one million permutations; bonobo vs. chimpanzee Bonferroni-corrected *P* value cutoff of 0.05/5 = 0.01; Sumatran vs. Bornean orangutan Bonferroni-corrected *P* value cutoff of 0.05/8 = 0.00625). Between bonobo and chimpanzee, each of the five gene families tested exhibited a significant difference in its copy number (*P* values of 1.67 × 10^−3^, <10^−6^, <10^−6^, <10^−6^, and <10^−6^, for *BPY2*, *CDY*, *DAZ*, *RBMY* and *TSPY* gene families, respectively). On the contrary, between the two orangutan species, we only found a significant difference in copy number for *XKRY* (*P* value = 1.32 × 10^−3^; see supplementary table S5, Supplementary Material online, for *P* values for the other gene families). Thus, significant differences in Y ampliconic gene copy number do exist, even between closely related species.

#### Accelerated Evolution Rates of Copy Number across Species

Building upon this observation, we tested whether copy number in Y ampliconic gene families is conserved across great ape species and identified species with significant gain or loss of gene copies. For this purpose, we used CAFE (Han et al. 2013), a tool that implements a stochastic birth-and-death process to model the expansion and contraction of gene family sizes over a phylogeny, and ran it with the Y-chromosome-specific phylogenetic tree (see Materials and Methods) and the median copy number per species for each of the families as input. We performed simulations to validate the use of CAFE for the given data set (see supplementary note 1, Supplementary Material online). CAFE estimated the rate of birth/death (*λ*) of Y-chromosome ampliconic genes in great apes as 0.05 events per million years, which is ∼21 times higher than the rate previously reported for non-Y gene families in the great ape ancestor (0.0024 per million years) (Hahn et al. 2007) and ∼31 times higher than the one inferred for non-Y gene families in TMRCA of mammals (0.0016 events per million years) (Demuth et al. 2006).

**Fig. 4.**
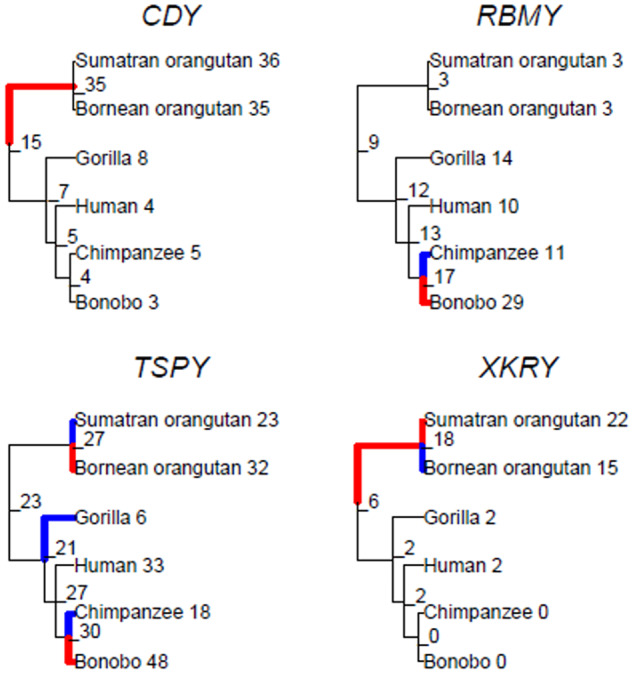
—Results of CAFE analysis identifying Y ampliconic gene families with significant shifts in gene copy number when compared with their ancestors. For each gene family with a significant difference in copy number, the phylogenetic tree representing the estimated copy number at internal nodes is shown. Significant shifts are highlighted in blue (contraction) and red (expansion). The copy numbers at the internal nodes were predicted by CAFE.

CAFE predicted that two of the nine Y ampliconic gene families tested had a significant expansion or contraction in their size (*RBMY*, *P *=* *0.001; *XKRY*, *P *=* *0.001; table 1 and fig. 4; Bonferroni-corrected *P* value cutoff of 0.05/9 = 0.006; nine gene families) and two additional gene families had low *P* values even if nonsignificant after correcting for multiple testing (*CDY*, *P *=* *0.018; *TSPY*, *P *=* *0.009; table 1 and fig. 4). For these four gene families, CAFE also provided *P* values for each branch of the phylogenetic tree with a significant gain or loss of gene copies when compared with its immediate ancestral node (Bonferroni-corrected *P* value cutoff of 0.05/10 = 0.005; ten nodes in great ape phylogenetic tree). Three interesting patterns emerged from this analysis (fig. 4 and supplementary table S6, Supplementary Material online). *First*, the *TSPY* gene family, which had consistently high variation in copy number across great apes (figs. 2 and 3), had the largest number of branches with significant differences in copy number across the phylogeny. We observed significant lineage-specific reductions in its family size in chimpanzee (from 30 copies inferred in the immediate ancestral node to 18 copies in chimpanzee, *P *=* *1.39 × 10^−9^), gorilla (from 21 to 6 copies, *P *=* *5.07 × 10^−5^), and Sumatran orangutan (from 27 to 23 copies, *P *=* *1.01 × 10^−3^), and significant expansions in Bornean orangutan (from 27 to 32 copies; *P *=* *2.68 × 10^−4^) and bonobo (from 30 to 48 copies, *P *=* *3.89 × 10^−7^). *Second*, two gene families (*CDY* and *XKRY*) showed significant expansions in the branch leading to the two orangutan species, and one of them (*XKRY*) also exhibited significant differences between the two orangutan species. In the case of *CDY*, the node connecting the two orangutan species gained a significant number of copies when compared with the common ancestor of great apes (from 15 to 35 copies, *P *=* *1.86 × 10^−3^). In the *XKRY* gene family, there was also a significant gain in gene copies in the common ancestor of Bornean and Sumatran orangutans when compared with the common ancestor of great apes (from 6 to 18 copies, *P *=* *4.31 × 10^−3^). Additionally, Bornean orangutan lost gene copies (from 18 to 15 copies, *P *=* *2.86 × 10^−3^) and Sumatran orangutan gained gene copies (from 18 to 22 copies; *P *=* *6.46 × 10^−4^) when compared with their common ancestor. And *third*, intriguingly, in the *RBMY* gene family, bonobo gained gene copies (from 17 to 29 copies, *P *=* *2.02 × 10^−7^), whereas chimpanzee lost gene copies (from 17 to 11 copies, *P *=* *9.61 × 10^−4^), when compared with their common ancestor.

**Table 1:** Differences in Y Ampliconic Gene Copy Numbers across Species as Evaluated with CAFE

Gene Family	CAFE *P* Value
*BPY*	0.345
*CDY*	0.014
*DAZ*	0.331
*HSFY*	0.488
*PRY*	0.209
*RBMY*	**0.001**
*TSPY*	0.008
*VCY* ^a^	0.187
*XKRY*	**0.001**

Note.— To determine which ampliconic gene families vary in their copy number and to identify significant expansions or contractions of gene family size across great apes, we performed CAFE analysis. Significant *P* values (Bonferroni-corrected *P* value cutoff of 0.05/9 = 0.006; nine gene families) are in bold. The *P* values for individual branches along the phylogenetic tree of great apes are available in supplementary table S6, Supplementary Material online, and gene losses and gains are shown in figure 4.

^a^ For *VCY*, power is limited because we only used the data from two species (chimpanzee and human). This gene family is absent in the other great ape species analyzed (see text for details).

To evaluate the influence of intraspecific variation on our analysis of gene copy number evolution, we ran CAFE while using the data for five randomly subsampled individuals (instead of a single summary value, i.e., median) per species (an approximately star phylogeny among individuals of the same species was assumed, see Materials and Methods). The sample size of five here corresponds to the smallest sample size we have per species (for Sumatran orangutan). This procedure was performed 100 times. We observed that differences in chimpanzee, bonobo, and gorilla lineages were still significant most of the time (in 83–100 times out of 100, supplementary table S7, Supplementary Material online). In the case of orangutans, the shift in *CDY* copy number was supported in 100 out of 100 replicates, and in the *TSPY* and *XKRY* gene families, the shift was supported in 12–68 out of 100 replicates (supplementary table S7, Supplementary Material online). Thus, intraspecific variability in the studied samples does not affect the robustness of our results, except for the orangutan-specific observations for the *TSPY* and *XKRY* families.

### Conservation of Y Ampliconic Gene Expression in Great Apes

#### Expression Levels of Y Ampliconic Gene Families

**Fig. 5.**
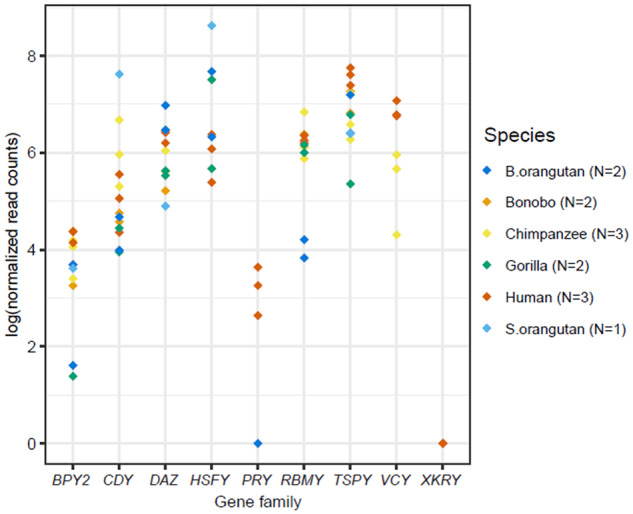
—Summary of gene expression levels across great apes. In the dot plot below, the *x* axis represents nine ampliconic gene families and the *y* axis represents their expression levels. The plot represents testis-specific expression of 12 great ape samples. Each dot within a gene family represents expression levels of an individual and the color of the dot denotes the species it belongs to. Missing dots represent gene families that are considered missing or pseudogenized, and their expression levels are excluded from the gene expression analysis (supplementary table S8, Supplementary Material online).

To study evolution of Y ampliconic gene expression, we evaluated expression levels for these gene families in great ape testis samples. We assembled complete or partial reference Y ampliconic gene transcripts using publicly available, or generated by us, RNA-Seq data sets (supplementary data set S3, Supplementary Material online) and used them to estimate the ampliconic gene expression across great apes (supplementary table S8, Supplementary Material online; see Materials and Methods). Namely, reference transcript data for bonobo were generated using RNA-Seq data sets produced in-house, for Bornean orangutan—using a mix of publicly available and generated in-house RNA-Seq data sets, whereas such data for other species were retrieved from publications (Brawand et al. 2011; Ardlie et al. 2015; Ruiz-Orera et al. 2015; Carelli et al. 2016; Fungtammasan et al. 2016; Tomaszkiewicz et al. 2016). The endangered status of great ape species posits a particular challenge for collection of tissues from these animals. Because of this hurdle, in this study, we were able to include only two to three sampled individuals per species (fig. 5; we excluded the results for Sumatran orangutan because only one sample was available for this species; see Materials and Methods).

Our analysis of Y ampliconic gene expression levels led to the following observations (fig. 5). The *BPY2*, *PRY*, and *XKRY* gene families had consistently low expression levels across great apes. In contrast, the *TSPY* and *HSFY* gene families had comparatively high expression levels across species, and intra- and interspecific variation in expression levels was also high for these two gene families. In comparison, the *CDY*, *DAZ*, and *RBMY* gene families had intermediate expression levels and limited intra- and interspecific variation. Surprisingly, in chimpanzee and human, the expression levels for the *VCY* family were higher than those for the *BPY2* and *CDY* gene families, even though the *VCY* family was lost in the majority of great apes, whereas the *BPY2* and *CDY* gene families were conserved across great apes (fig. 1*A*).

#### Evolution of Gene Expression

Using this data set (fig. 5 and supplementary table S8, Supplementary Material online), we next evaluated whether expression levels of the Y ampliconic gene families were conserved across great ape species. To examine this, we performed phylogenetic ANOVA, which conducts an ANOVA-like test while taking the phylogenetic relationship of great apes into consideration (see Materials and Methods). This test was carried out for five gene families—*BPY2*, *CDY*, *DAZ*, *RBMY*, and *TSPY*—which are present in all the great ape species analyzed (fig. 1*A*). Phylogenetic ANOVA was performed via applying the EVE model (Rohlfs and Nielsen 2015) separately to each of the five Y ampliconic gene families and identified that all five of them had conserved expression, that is, with no branches experiencing significant speedup or slowdown in expression evolution, across great apes (supplementary table S9, Supplementary Material online). To test the validity of our conclusions given the small sample size and particular gene copy numbers, we performed simulations for different parameters under the EVE model and generated gene expression levels with the sample sizes identical to those in our study (supplemental note 2, Supplementary Material online). In 95 out of 100 simulations, we were able to predict conservation of gene expression correctly.

### The Relationship between Copy Number and Gene Expression

**Fig. 6.**
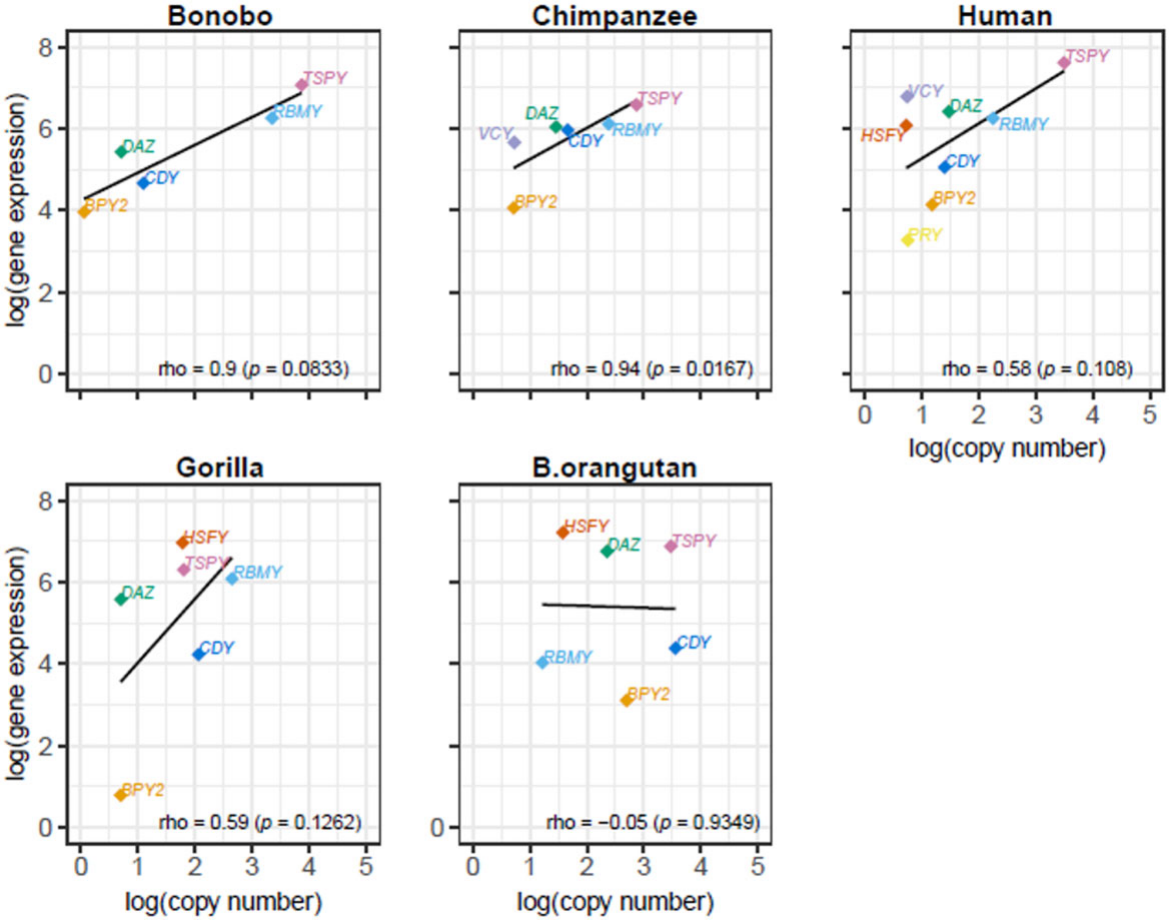
—Relationship between copy number and gene expression of Y ampliconic gene families in great ape species. The five scatter plots represent the relationship between expression and copy number for each of the five species, and the name of the species is present at the top of each plot. In each of the scatter plots, the *x* axis represents natural logarithm of median copy number and the *y* axis represents natural logarithm of median gene expression. The Spearman correlations were calculated using the cor.test() function in R and the *P* values are in parentheses. The black line is the linear function fitted to the given data points. The dots are color coded to represent the nine gene families, with missing dots corresponding to the gene families that are pseudogenized, deleted, or not expressed, in that species.

We studied the relationship between Y ampliconic gene copy number and expression levels across five great ape species (with Sumatran orangutan excluded due to the lack of multiple testis samples). When we analyzed the median copy number of each family from our copy number data set (fig. 2) together with the median gene expression levels of these families from our gene expression data set (fig. 5), we observed that the correlation between them for the majority of species was positive, consistent with previous results in humans (Vegesna et al. 2019). However, there were also some differences (fig. 6). In bonobo and chimpanzee, we identified a strong positive correlation between gene copy number and their expression levels across gene families (bonobo: Spearman correlation *ρ *=* *0.9, *P *=* *0.083; chimpanzee: *ρ *=* *0.94, *P *=* *0.017). In human and gorilla, we also observed a positive correlation, but it was weaker (human: *ρ *=* *0.58, *P *=* *0.108; gorilla: *ρ *=* *0.59, *P *=* *0.126). In the case of Bornean orangutan, we did not observe a positive correlation (*ρ *=* *−0.05, *P *=* *0.93), and one of the reasons that might explain this finding is the high variation in Y ampliconic gene copy number in this species (fig. 3).

**Fig. 7.**
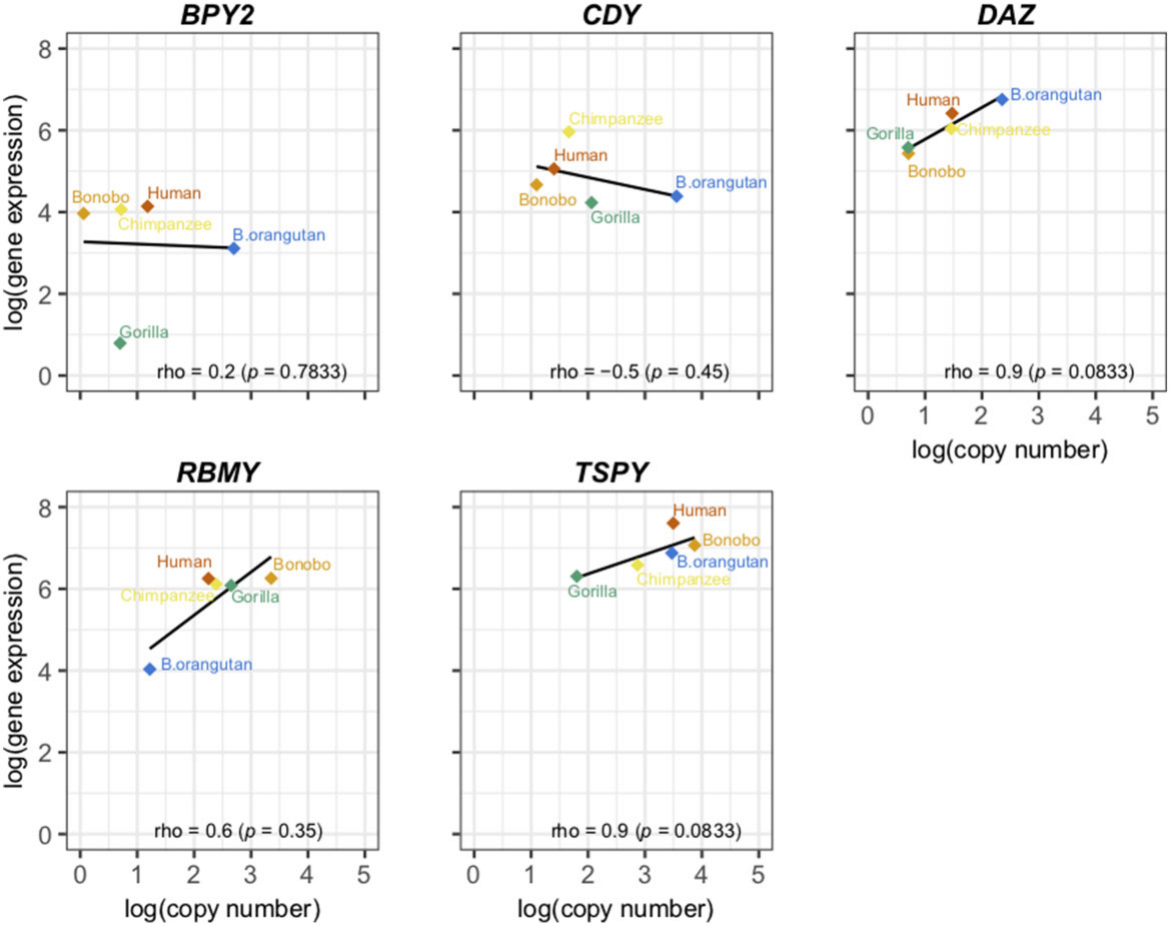
—Relationship between copy number and gene expression across species. In each of the scatter plots, the *x* axis represents natural logarithm of median copy number and the *y* axis represents natural logarithm of median gene expression. The Spearman correlations were calculated using the *cor.test()* function in R and the *P* values are in parentheses. The black line represents the linear function fitted to the given data points. The dots are color coded to represent the five species. The five scatter plots represent the relationship between expression and copy number for each of the five gene families, with the name of the gene family present at the top of each plot. Only the gene families that are present in all species are shown here.

Next, we studied the relationship between copy number and gene expression separately for each gene family (fig. 7). Previous studies in humans showed that within a Y ampliconic gene family the variation in gene copy number does not correlate with their gene expression (Vegesna et al. 2019). Here, we tested whether the longer divergence times between great ape species enabled gene copy number to influence expression levels. We observed that, across species, there was a strong (but statistically nonsignificant) positive correlation of gene expression level with gene count for *DAZ* (*ρ *=* *0.9, *P *=* *0.083) and *TSPY* (*ρ *=* *0.9, *P *=* *0.083), and a moderate and nonsignificant correlation for *RBMY* (*ρ *=* *0.6, *P *=* *0.35). There was no such trend observed for *BPY2* (*ρ *=* *0.2, *P *=* *0.783) with all species having similar expression levels except for gorilla, which had comparatively low expression levels. A negative but nonsignificant relationship was observed for *CDY* (*ρ *=* *−0.5, *P *=* *0.45), with chimpanzee and human having higher expression levels but fewer gene copies in comparison to gorilla and Bornean orangutan. In general, we might be lacking power to detect significant associations between gene expression levels and copy number of Y ampliconic genes because of the small sample size. However, we did observe a trend of copy number influencing gene expression levels in three out of five gene families tested.

In the analyses above (figs. 5–7), human Y ampliconic transcript sequences were added to the reference, to which species-specific RNA-Seq reads were mapped, to alleviate the potential limitation arising due to some species-specific transcripts being incomplete. Because adding human transcripts to the reference could have introduced reference bias, we repeated the same analyses while excluding human transcripts from the reference. The resulting ampliconic gene expression levels and their correlations with copy number exhibited similar trends (supplementary figs. S7–S10, Supplementary Material online) to those observed when human reference was included (figs. 5–7), with the only difference being a negative (but still nonsignificant) correlation of gene expression level with gene count for *DAZ* when human reference was excluded (supplementary fig. S10, Supplementary Material online).

## Discussion

To study evolution of multicopy gene families on the Y chromosome in the extant great apes, we analyzed copy number and expression levels of Y ampliconic genes across six great ape species. For the first time, we estimated variation in copy number of Y ampliconic gene families in a large sample of bonobos (previously only two individuals were compared [Oetjens et al. 2016]) and in two species of orangutan. We also generated testis expression data for Y ampliconic gene families for bonobo and Bornean orangutans, thus providing unique data sets that were previously missing from publicly available resources. Combining these new and previously published data, we investigated the evolution of Y ampliconic gene copy number and of their expression levels as well as the evolutionary relationship between them. Some of our tests showed strong trends but lacked statistical significance, likely because they were underpowered due to a small sample size. Increasing the sample size for this type of study is currently extremely challenging because of the limited availability of samples from endangered species.

### Loss of Some Y Ampliconic Gene Families in All Nonhuman Great Ape Species Examined

Combining data from this and other studies (Skaletsky et al. 2003; Hughes et al. 2010; Cortez et al. 2014; Oetjens et al. 2016; Tomaszkiewicz et al. 2016), we have demonstrated that, compared with human, all other great ape species examined lack one (*VCY* missing in gorilla and both species of orangutan) or several (*HSFY*, *PRY*, and *XKRY* pseudogenized in bonobo and chimpanzee) Y ampliconic gene families (fig. 1*A*). We discovered that gene families with low copy number in some species are frequently lost or pseudogenized in other species. For instance, we found that *PRY* and *XKRY*, which were pseudogenized in bonobo and chimpanzee, have low copy number in other great apes examined. Similarly, a recent study from our group showed that Y ampliconic gene families with low copy numbers in humans (Vegesna et al. 2019) were pseudogenized or lost in some great ape species (*HSFY*, *PRY*, *VCY*, and *XKRY*) due to the lack of recombination, Muller’s ratchet, and/or nonallelic homologous recombination on the Y. Some of these genes could have lost their function and could be dispensable, could have been replaced by closely related genes, or could have moved to another location in the genome. Regardless of the mechanism, it is a fact that not all human Y ampliconic gene families are essential in all great ape species.

Our study indicates that low expression levels could also be an important predictor of gene family’s nonessentiality on the Y. Consistent with this hypothesis, lowly expressed *PRY* and *XKRY* gene families were pseudogenized in chimpanzee and bonobo. However, gene families such as *VCY* and *HSFY*, which were also lost in several great ape species, had relatively high expression levels. The essentiality and expression of Y ampliconic gene families should be studied in conjunction with that of their autosomal and X-chromosomal paralogs in future studies. *VCY* and its paralog *VCX*, which is present on the X chromosome, are highly similar in sequence (at least in humans), and thus the loss of *VCY* in some species could be compensated by *VCX* (Vegesna et al. 2019). *HSFY* could have undergone neofunctionalization in humans (Vegesna et al. 2019). Therefore, in the great ape species that lost *HSFY*, its X-chromosome paralogs (*HSFX1* and *HSFX2*) could also compensate for its ancestral function. Interestingly, the X-linked paralogs of the *HSFY* (*HSFX1* and *HSFX2*), *RBMY* (*RBMX* and *RBMX2*), and *VCY* (*VCX*, *VCX2*, *VCX3A*, and *VCX3B*) are present in a multicopy state on the human X chromosome. However, regions with high copy number variation on the X chromosome do not include Y ampliconic gene paralogs (Lucotte et al. 2018). There is a need to study the relationship between variation in ampliconic gene copy number on the X and Y chromosomes in the future.

### Y Ampliconic Gene Families: Life on Palindromes and Tandem Repeats

We observed a positive relationship between copy number and its variance for Y ampliconic gene families in great apes. This finding echoes recent studies in humans (Ye et al. 2018; Vegesna et al. 2019) and points toward a similar organization of these gene families in repeats across great ape species and in their common ancestor. In human and chimpanzee, most Y ampliconic gene families (except for *TSPY*, which is organized as a tandem repeat) are located in palindromes (Skaletsky et al. 2003; Hughes et al. 2010). Palindromes also exist on the gorilla Y chromosome (Tomaszkiewicz et al. 2016). High copy number variation in ampliconic genes in bonobo and orangutans found here suggest that their Y chromosomes also have repetitive structure, confirming cytogenetic findings (Gläser et al. 1998).

The presence of palindromes on the Y chromosome in great apes enables frequent rearrangements via nonallelic homologous recombination and gene conversion among different palindrome arms, leading to the observed high variation within species. Gene conversion leads to conservation of gene sequences and their rescue from accumulation of deleterious mutations. A study of palindrome P8 in healthy humans showed diverse palindromic structures carrying from one to four copies of *VCY* (Shi, Massaia, et al. 2019). Large-scale chromosomal rearrangements also contribute to the dynamic copy number evolution of Y ampliconic gene families across great apes, as shown by previous cytogenetic analyses (Gläser et al. 1998; Repping et al. 2006; Shi, Louzada, et al. 2019), as well as by an example in the next paragraph.

Interesting patterns were observed for median copy numbers for each of the five Y ampliconic gene families present in bonobo and chimpanzee (fig. 3). For low-copy-number families, we found a total of six gene copies (one *BPY2*, three *CDY*, and two *DAZ* gene copies) in bonobo. For the same gene families in chimpanzee, six gene copies (one *BPY2*, three *CDY*, and two *DAZ* gene copies) are present on three palindromes of the short arm and five gene copies (one *BPY2*, two *CDY*, and two *DAZ* genes) are located on the three palindromes of the long arm (Hughes et al. 2010). The differences in copy number between bonobo and chimpanzee are consistent with a deletion of the three palindromes bearing five gene copies on the long Y arm in the bonobo lineage after its divergence from the bonobo–chimpanzee common ancestor. This hypothesis is strengthened by the results of a cytogenetic study that mapped the *CDY* and *DAZ* gene families to the short arm of the bonobo Y, but to both short and long arms of the chimpanzee Y (Schaller et al. 2010). This pattern was in contrast to that observed for high-copy-number gene families, *TSPY* and *RBMY* (fig. 3). We found that bonobo Y had approximately three times more *TSPY* and *RBMY* gene copies than the chimpanzee Y (supplementary table S4, Supplementary Material online). Consistent with this finding, a cytogenetic study reported high amplification of *RBMY* and *TSPY* gene families via segmental duplications of a large euchromatic segment in bonobo (Gläser et al. 1998).

### Evolutionary Forces Affecting Copy Number Variation among Great Apes

Our test of conservation of gene copy number across great ape species indicated significant differences in copy numbers of *CDY*, *RBMY*, *TSPY*, and *XKRY*. Chimpanzee had lost, whereas bonobo had gained, *TSPY* and *RBMY* gene copies when compared with their common ancestor. In the case of gorilla, we observed a significant loss of *TSPY* gene copies. Additionally, interspecific differences in copy number in our data set were correlated with interspecific differences in copy number variance (fig. 3). What can explain the differences observed among species?

Whereas a detailed analysis is outside the scope of our study, we can speculate about the evolutionary forces driving copy number variation of Y ampliconic gene families in great apes. If a certain range of copy number were beneficial, then directional selection would limit variation in copy number even for large gene families. We did not observe such a pattern. In contrast, if variability in copy numbers were beneficial, then diversifying selection would enhance variation in copy number even for small gene families. This pattern was also not found. Selection might be operating within certain great ape species, or at particular gene families. Sperm competition could be the selective force behind Y ampliconic gene number evolution; however, the small number of great ape species available precludes us from a rigorous statistical analysis of this relationship. Note that high variability observed for copious gene families might mask signatures of diversifying selection, and vice versa, low variability observed for gene families with low copy number might mask signatures of directional selection. Accelerated gains of ampliconic gene copies might facilitate the introduction of new mutations and increase adaptation, which should be tested in a separate study addressing both copy number and sequence evolution of individual gene copies simultaneously.

We observed that variance in copy number was approximately proportional to the size of gene family (fig. 3), suggesting that larger families have more opportunities for rearrangements resulting in high-copy-number variability. Thus, our data overall are consistent with random genetic drift resulting from frequent copy number changes of repetitive regions being the major driver of Y ampliconic gene families’ evolution. A similar conclusion was reached when a larger data set of humans representing multiple haplogroups was studied (Ye et al. 2018). Moreover, though our CAFE analyses revealed significant bursts in gains/losses of copy numbers across specific lineages in *CDY*, *RBMY*, *TSPY*, and *XKRY* (fig. 4), we note that these results could be the effect of genetic drift as the phylogenetic tree relating great apes that was used as input to CAFE had time measured in thousands of years rather than in an evolutionary time unit such as coalescent time, which would intrinsically account for both time in generations and effective size.

### Evolution of Gene Expression, and the Relationship between Gene Expression and Copy Number

Our analysis suggests that the Y ampliconic gene families present in all great ape species studied exhibit limited interspecific variation in gene expression, and this variation was not significant with our EVE model analysis, either due to the small sample size or reflecting overall conserved expression levels for these gene families. A larger study is needed to distinguish between these two possibilities. If interspecific variation is indicated in such a study, then it would echo an earlier study, in which genome-wide variation in gene expression across different tissues in human and chimpanzee was investigated, and genes with high intraspecific variation were found to exhibit high interspecific variation, particularly in testis (Khaitovich et al. 2006, 2005). If, in contrast, conserved expression levels are confirmed, then DNA methylation might play an important role in dosage regulation of duplicated genes (Chang and Liao 2012), and future studies should investigate the upstream regions of Y ampliconic genes for DNA methylation patterns.

Even though we have found conservation of gene expression regardless of gene copy number, there are several factors that could have contributed to the lack of correlation between these two measurements. First, it is possible that we have overestimated the number of functional copies within a family because some pseudogenes could have been captured together with functional genes. Second, incomplete transcripts might have contributed to our estimates of gene expression levels. Third, considering expression levels from two to three individuals different from the ones used to estimate copy number might have also contributed to the lack in precision of our gene expression estimates. Additional studies including a large number of individuals, from which both copy number and gene expression will be measured, should clarify this relationship.

In humans, testis tissue tolerates high variation in gene expression levels and undergoes dosage regulation to maintain overall conserved gene expression in the presence of gene copy number variation (Vegesna et al. 2019). However, across species, we observed a mixed pattern in the relationship between gene expression levels and copy number: *DAZ*, *RBMY*, and *TSPY* showed a positive correlation, *BPY2* displayed no association, and *CDY* had a negative correlation (fig. 7). These results imply that, given enough evolutionary time, copy number could influence gene expression levels in some ampliconic gene families.

We observed a positive correlation between copy number and gene expression across gene families in each great ape species but Bornean orangutan. This correlation was stronger in bonobo and chimpanzee, species experiencing high levels of sperm competition, suggesting that in these species it can be particularly important biologically. In general, evolution of copy number and gene expression, although related to each other, might follow different time scales. Our results suggest that evolution of copy number is faster than evolution of gene expression. Rapid, back-and-forth changes in copy number for Y ampliconic genes eventually influence the direction in which gene expression levels shift over longer periods of time.

It is important to note that factors such as age and cellular composition of the testis tissues could influence the estimated expression levels of the Y ampliconic gene families. These factors have to be addressed in future studies. Also, further refinements of reference transcriptomes for each species will aid in obtaining more accurate estimates of expression levels. Future studies should decipher isoform sequences of Y ampliconic genes, as well as of their X-chromosomal and autosomal counterparts, and analyze their differential expression, and thus will examine the dynamic evolution of male fertility genes in greater detail.

## Conclusions

Here we presented the first study exploring variation in copy number and expression of Y ampliconic genes across most great ape species (only omitting Tapanuli orangutan, which was recently discovered). To evaluate copy number of Y ampliconic genes, we used ddPCR assays, which were previously demonstrated to be highly accurate and reproducible (Hindson et al. 2013; Taylor et al. 2017; Vegesna et al. 2019). We presented ampliconic gene copy number variation in bonobos and orangutans for the first time and showed that orangutans have the highest copy number and the highest variation in copy number across great apes. We observed significant differences in copy number in four out of nine Y ampliconic gene families. To obtain the gene expression data set, we assembled transcripts and estimated expression levels of Y ampliconic genes using publicly available and generated in-house testis-specific RNA-Seq data sets. The analysis of this data set indicated conserved evolution, that is, none of the Y ampliconic gene families had significant shifts in their expression levels between species, despite substantial and significant variation in their copy number. We observed a positive correlation between copy number and expression levels for the *DAZ*, *RBMY*, and *TSPY* gene families, in contrast to the results in human, where such correlation was not observed for any Y ampliconic gene families (Vegesna et al. 2019). Thus, copy number can influence gene expression given sufficient evolutionary time. Our results have important implications for understanding Y-chromosome evolution in endangered great apes.

## Availability of Data and Materials

Primer sequences are presented in supplementary data set S1, Supplementary Material online. ddPCR replicates of copy numbers are shown in supplementary data set S2, Supplementary Material online. Transcript sequences are presented in supplementary data set S3, Supplementary Material online; expression data are presented in supplementary table S8, Supplementary Material online. Code used in the manuscript is available at github link: https://github.com/makovalab-psu/Y-AmpGene_CN_GE_GreatApes.git.

## Supplementary Material

evaa088_Supplementary_Data
