# Supplementary material for: Ampliconic Genes on the Great Ape Y Chromosomes: Rapid Evolution of Copy Number but Conservation of Expression Levels
Source: Genome Biol Evol. 2020 May 6;12(6):842–59. doi: 10.1093/gbe/evaa088 (PMC7313670; doi:10.1093/gbe/evaa088)
Supplement: evaa088_Supplementary_Data [file evaa088_supplementary_data.zip › Supp_Notes_Tables_Figures_Great_ape_CN_expression-Old.pdf]

Supplemental Materials

Supplemental Note 1. CAFE Simulations **3**

Supplemental Note 2. EVE Simulations **8**

Supplemental Tables **13**

Table S1. All the great ape copy number samples used in the study. 13

Table S2. List of RNA-Seq samples. 15

Table S3. Mean values of ampliconic gene copy numbers across at least three ddPCR replicates. 16

Table S4. Summary of ampliconic gene copy numbers across great apes. 18

Table S5. P-values from permutation tests for copy number differences between Sumatran and Bornean orangutans. 20

Table S6. The branch-level p-values showing the presence of significant shift in copy number when compared to its immediate ancestor in the great ape phylogenetic tree. 21

Table S7. Summary of CAFE results with five individuals per species added as star phylogeny. 22

Table S8. Gene expression values for Y ampliconic gene families across great apes. 23

Table S9. EVE-model-based likelihood ratios and p-values showing no significant shift in gene expression of shared ampliconic gene families across great apes. 24

Supplemental Figures **25**

Figure S1. Transcriptome assembly pipeline. 25

Figure S2. Heatmap depicting the Euclidean distances of gene expression values between pairs of sampled individuals. 26

Figure S3. Principal components analysis of great ape gene expression values (RLD-normalized). 27

Figure S4. Principal components analysis of great ape gene expression values (VST-normalized). 28

Figure S5. Principal components analysis of the overall copy number of Y ampliconic gene families across great apes. 29

Figure S6. Across species, gene families with higher copy number have higher variance. 30

Figure S7. Heatmap depicting the Euclidean distances of gene expression values (using assembled species-specific transcripts as a reference) between pairs of sampled individuals. 31

Figure S8. Summary of gene expression levels across great apes (using assembled species-specific transcripts as a reference). 32

Figure S9. Relationship between copy number and gene expression (using assembled species-specific transcripts as a reference) of Y ampliconic gene families in great ape species. 33

Figure S10. Relationship between copy number and gene expression (using assembled species-specific transcripts as a reference) across species. 34

References **35**

Supplemental Note 1. CAFE Simulations

Simulations to test gene family size. The great ape dataset includes copy number estimates of nine gene families from six species. We tested whether nine gene families (*n*=9) were sufficient to predict the rate of gene birth and death, because uncertainty in the rate parameter could influence *p*-value estimates generated by CAFE (Han et al. 2013). To test this, we used the gene family copy numbers and the phylogenetic tree from the original CAFE article (Hahn et al. 2007). However, we only used primate-specific data (human, chimpanzee, and macaque) in our simulations. Our goal was to test the reproducibility of birth and death rate (λ) predictions by CAFE. First, we fixed the phylogenetic tree representing the three primate species to be used in all simulations as (((Chimp:6,Human:6):18,Macaque:24)), and then applied different combinations of gene family copy numbers as input to estimate λ. As a filtering step, we removed all the gene families that had >200 gene copies cumulatively across the three species. This filtering step was used to remove excessively large gene families and retain gene families whose copy numbers are in the range observed for Y chromosome ampliconic gene families. Next, we filtered all gene families that had the same gene count in all species to ensure there was variation across the species. These filtering steps reduced the gene family count from ~9,800 to 2,445. From this set we picked 30 gene families uniformly at random, and applied CAFE on each to estimate λ. We repeated this step 20 times (Table SN1). Next, from each set of 30 gene families, we subsampled 5, 10, and 15 gene families uniformly at random and applied CAFE on each to estimate λ. We observed that with different sizes and combinations of gene families CAFE predicted different values for λ (Table SN1). Next, we picked 100 gene families with highest variation in copy number across species and performed the same analyses as above. For all input sizes of 5, 10, 15, and 30 considered, the estimated rate of gene birth and death was identical (λ=0.041667; Table SN2). This result suggests that CAFE requires gene families with high variation in their gene count across species to predict a consistent λ value, and the majority of the ampliconic genes considered here have high variation in gene count across great ape species, thereby making CAFE an ideal approach to study their evolution.

Simulations to include multiple samples per species in CAFE analyses. CAFE typically takes the mean/median value as a representation of copy number of gene families in a species.

However, we observed high intraspecific variation in copy number of gene families within our dataset, and wished to leverage this information in our predictions of gene family copy number evolution. We therefore tested whether CAFE could reproduce the significant differences observed in great ape ampliconic gene families when we provide it with multiple individuals per species instead of the default application of a single value (mean or median copy number) per gene family as input. With the presence of multiple samples per species, we assumed that CAFE might take the variation in copy number into consideration while estimating *p*-values. Because the exact phylogenetic relationship among the samples is unknown in terms of the time since their common ancestor, we estimated the time since the most recent common ancestor (TMRCA) for each species using an external dataset (Hallast et al. 2016). We followed the same steps used in the generation of the phylogenetic tree of great apes (see Materials and Methods), except that here we calculated the TMRCA for all the combinations of samples available, and took the median TMRCA value within a species as the representative TMRCA for the species.

For each species, we added five branches at the tips (i.e. approximately a star phylogeny) of the great ape phylogenetic tree, such that each species was represented by five individuals (Supplementary Figure SA). The lengths of these five branches are represented by the TMRCA of lineages within each species, with a difference of one thousand years between each internal node. That is, the last split for each individual within a species was within four thousand years since the TMRCA of all five individuals, and this mimics a star phylogeny as the TMRCA ranging from 75 to 550 thousand years is much larger than four thousand years. From our dataset, we picked five individuals per species uniformly at random and provided their ampliconic gene family copy numbers as input to CAFE along with the updated phylogenetic trees (with star phylogenies at the tips) to test for significant gains or losses in copy number. This procedure was repeated 100 times. In these simulations, for each gene family we looked for significant shifts (gains or losses) in gene copy number at each branch along the phylogenetic tree, which is indicated by a *p*-value (Bonferroni-corrected *p*-value < 0.005; 0.05/10 by correcting for the 10 external and ancestral branches present on the phylogenetic tree) and compared them to the original run (Table S7) of CAFE. All significant observations identified in the analysis based on median copy number were also significant when we used multiple samples per species. However, within the set of 100 simulated replicates, the frequency in which each branch has a significant shift was not the same. On the ancestral orangutan branch, the *CDY* gene family

displayed a significant shift in all 100 simulations. On the bonobo and chimpanzee branches, the *RBMY* gene family showed a significant shift in 100 and 83 simulations, respectively. The *TSPY* gene family had significant shifts on multiple branches, with 99 simulations showing significance in bonobo and chimpanzee, 84 in gorilla, 57 in Bornean orangutan, and 39 in Sumatran orangutan. Finally, the *XKRY* gene family had 55 simulations with a shift in the ancestral branch of orangutans and 68 with a shift in Sumatran orangutan. The *XKRY*-specific shift in Bornean orangutan was significant only 12 times out of 100 simulations.

Table SN1. λ values based on randomly selected gene families. Each column indicates the number of genes used to estimate the rate of gene birth or death and each row represents different replicates.

N=5	N=10	N=15	N=30
0.011158	0.041667	0.034038	0.017218
0.035354	0.035568	0.022321	0.013378
0.003766	0.010327	0.009695	0.011261
0.007554	0.027215	0.0206	0.017613
0.041667	0.024575	0.017532	0.012252
0.007613	0.00628	0.008843	0.010396
0.006239	0.006899	0.011568	0.010448
0.026245	0.013568	0.041667	0.038559
0.041667	0.041667	0.041667	0.023911
0.004424	0.005405	0.006028	0.009473
0.013036	0.009137	0.012186	0.011632
0.009044	0.010184	0.00975	0.010824
0.004717	0.007606	0.008536	0.023141
0.041667	0.041667	0.032196	0.018089
0.01187	0.011803	0.010866	0.012213
0.004724	0.005352	0.008868	0.009709
0.005236	0.014412	0.010689	0.017878
0.041667	0.041667	0.036225	0.038344
0.004755	0.01066	0.012642	0.009939
0.017927	0.018403	0.020957	0.016235

Table SN2. λ values based on gene families with high copy number variation across species. Each column indicates the number of genes used to estimate the rate of gene birth or death and each row represents different replicates.

N=5	N=10	N=15	N=30
0.031804	0.041667	0.041667	0.041667
0.041667	0.041667	0.041667	0.041667
0.041667	0.041667	0.041667	0.041667
0.041667	0.041667	0.041667	0.041667
0.041667	0.041667	0.041667	0.041667
0.041667	0.041667	0.041667	0.041667
0.041667	0.041667	0.041667	0.041667
0.041667	0.041667	0.041667	0.041667
0.041667	0.041667	0.041667	0.041667
0.041667	0.041667	0.041667	0.041667

Supplemental Figure SA. The great ape phylogenetic tree with five individuals sampled per species (star phylogeny) used in CAFE analysis. Significant shifts were assessed for each of the internal edges numbered 1-10 (edges from the original phylogenetic tree), whereas shifts were not examined for external edges representing the star phylogenies relating sampled individuals within a species.

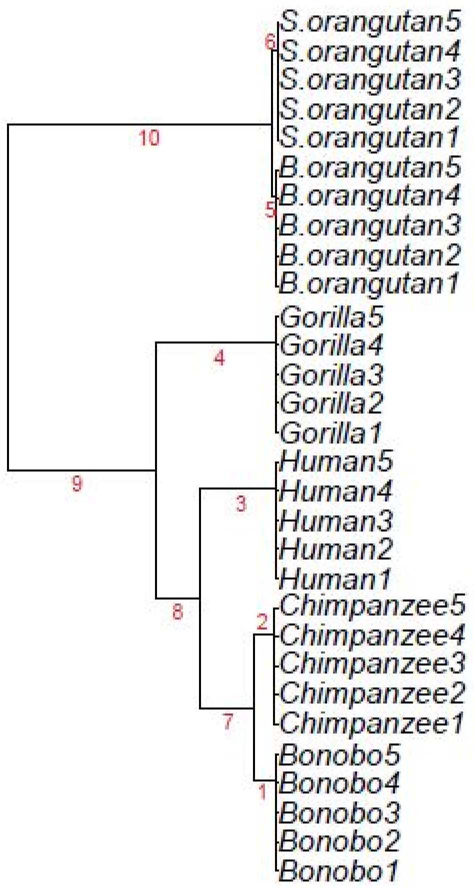

Supplemental Note 2. EVE Simulations

With the difficulty in obtaining testis samples to generate expression data, we had a small sample size for each species except for humans. Currently, the EVE model (Rohlfs & Nielsen 2015) cannot handle missing values, and so we limited our analysis to the five gene families that are found in all great ape species. In our dataset, we have two to three individuals sampled per species, except for humans in which we had testis expression measured for more than one hundred sampled individuals. To ensure that sample sizes were comparable across the sampled great apes, we used three (maximum size in non-human species) human testis expression samples that were sampled uniformly at random from the set with African Y-specific haplogroup E from the GTEx dataset (Ardlie et al. 2015). We chose samples with African ancestry as they are expected to have high genetic variation in humans. On the whole, we had 12 testis samples in total (three human, two bonobo, three chimpanzee, two gorilla, and two B. orangutan). We tested whether the EVE model performs well with five gene families and two or three individuals sampled per species.

The built-in function -H in the EVE model simulates expression values when provided with a phylogenetic tree, the number of individuals sampled per species, along with the parameter values for selection strength (α), drift (σ^2), ratio of within- to between-species variance (β), and optimal expression value (θ). The within species variance (τ^2) or population variance is represented by $\beta\frac{\sigma^2}{2\alpha}$ (Rohlfs et al. 2014). For the simulations, we matched sample sizes to empirical sample sizes (three, two, three, two, and two for human, bonobo, chimpanzee, gorilla, and B. orangutan, respectively), employed the great ape phylogenetic tree generated using whole-genome sequencing data (Locke et al. 2011), and considered the parameter values from the simulations studies presented in the EVE model article (Rohlfs & Nielsen 2014) (σ^2=5, α=3.0, β=6, and θ=100).

Using -H mode in the EVE model, we simulated gene expression values by varying one of the four parameter values (σ^2=5, α=3.0, τ^2=6, and θ=100) from 0.5 to 60 (0.5, 1, 3, 5, 10, 15, 20, 40, 60) at a time while keeping the other three parameters, sample size, and phylogenetic tree fixed. Based on the combination of parameters, the EVE model outputs an expression value matrix of size *m* x *n*, where *m* is the number of sampled expressed genes (*m*=5) and *n* is the

total number of individuals sampled across all species together (n=12). Using this matrix as input, it tests whether $\beta_i=\beta_{shared}$ for each simulated gene i, i=1,2,...,m. For each combination of parameters, we simulated gene expression values for 100 independent replicates. Because the parameters for the model were predefined and we did not introduce external variation, we expect the EVE model to predict that all of the simulated gene expression values are conserved across species and that the p-values for the tests of whether $\beta_i=\beta_{shared}$, should be non-significant (i.e. $p>0.05$). If the number of genes and sample sizes are insufficient, then we expect the EVE model to predict that expression of some or all genes is significantly different from the shared expression. Using the significance cutoff 0.05, we summarized the number of times a gene was differentially expressed across great apes in the 100 replicates of each combination of parameters. We plotted the percentage of replicates that have their p-value above the threshold of 0.05 as a heatmap (Figures SB-SD). We observed that 95% of replicates had a non-significant p-value and 5% of replicates could result from random sampling of expression values from a multinormal distribution by EVE model. Based on our simulation results, we are confident that for our sample sizes, the EVE model can predict the difference in gene expression variance correctly 95 out of 100 times.

Supplemental Figure SB. Proportion of replicates that have their *p*-value above the threshold of 0.05 when the selection (α) parameter varied from 0.5 to 60 while other parameters were fixed (σ^2=5, β=6, and θ=100).

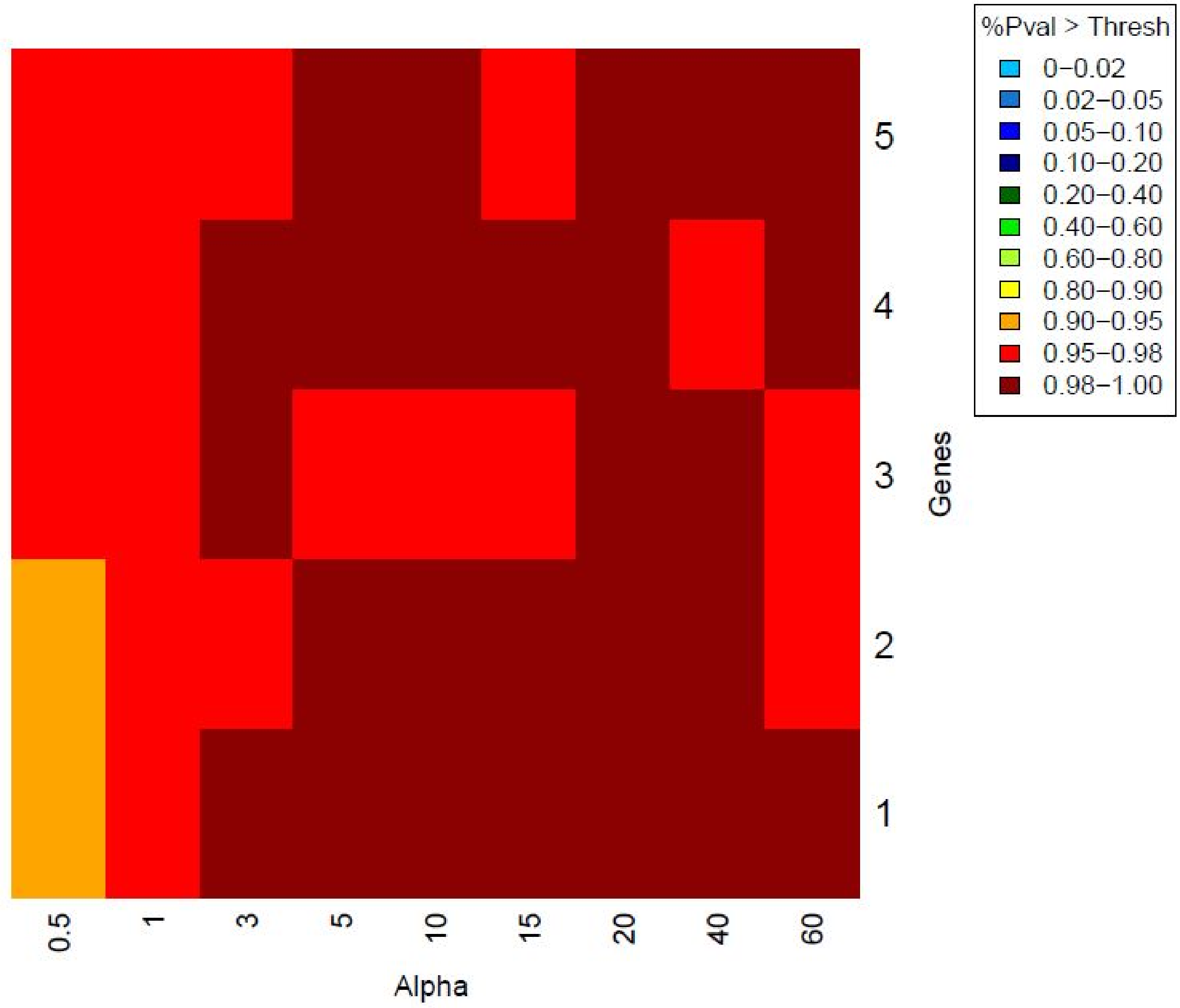

Supplemental Figure SC. Proportion of replicates that have their *p*-value above the threshold of 0.05 when the drift (σ^2) parameter varied from 0.5 to 60 while other parameters were fixed (α=3.0, β=6, and θ=100).

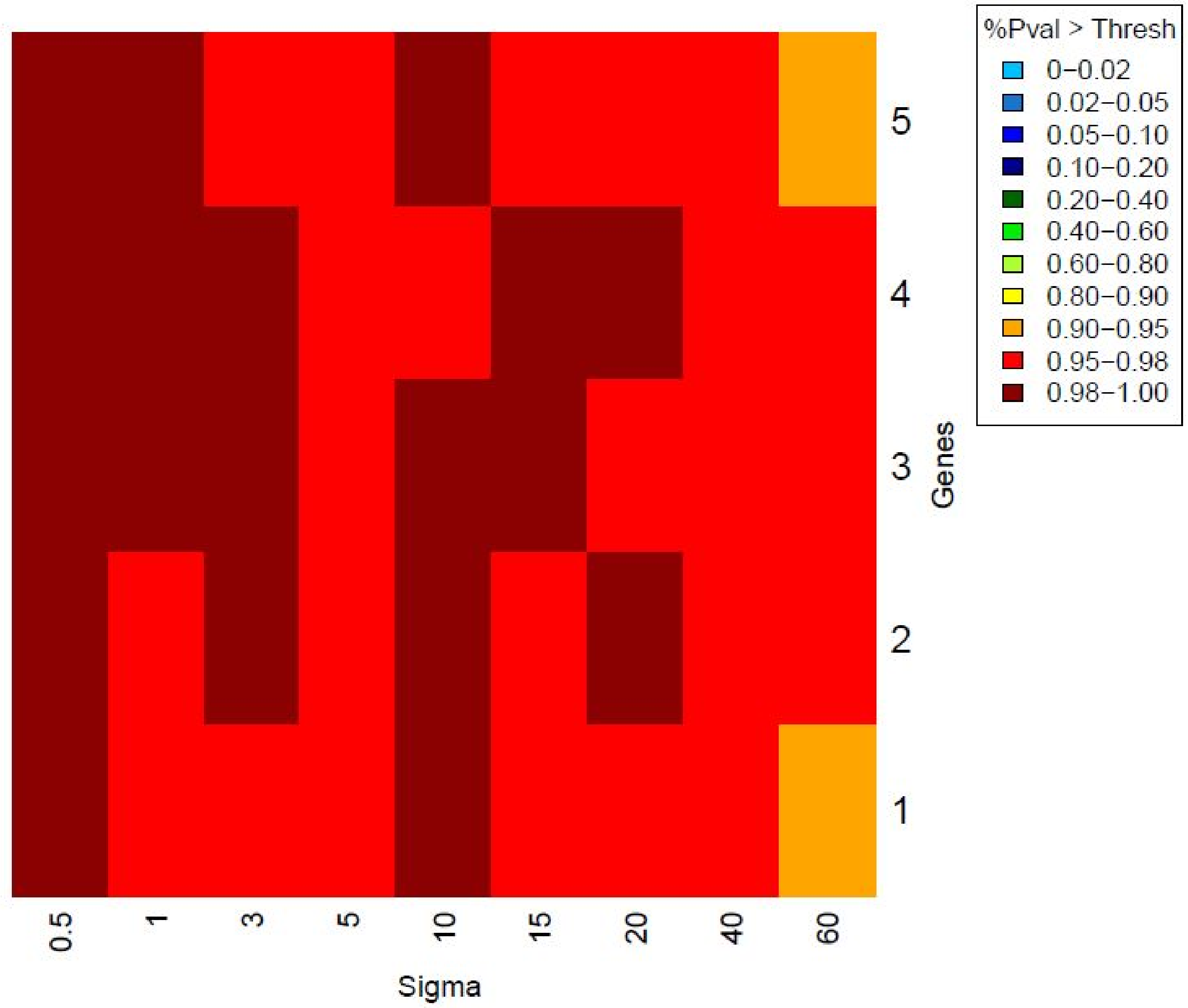

Supplemental Figure SD. Proportion of replicates that have their *p*-value above the threshold of 0.05 when the variation within species parameter (τ^2) varied from 0.5 to 60 while other parameters were fixed (σ^2=5, α=3.0, and θ=100).

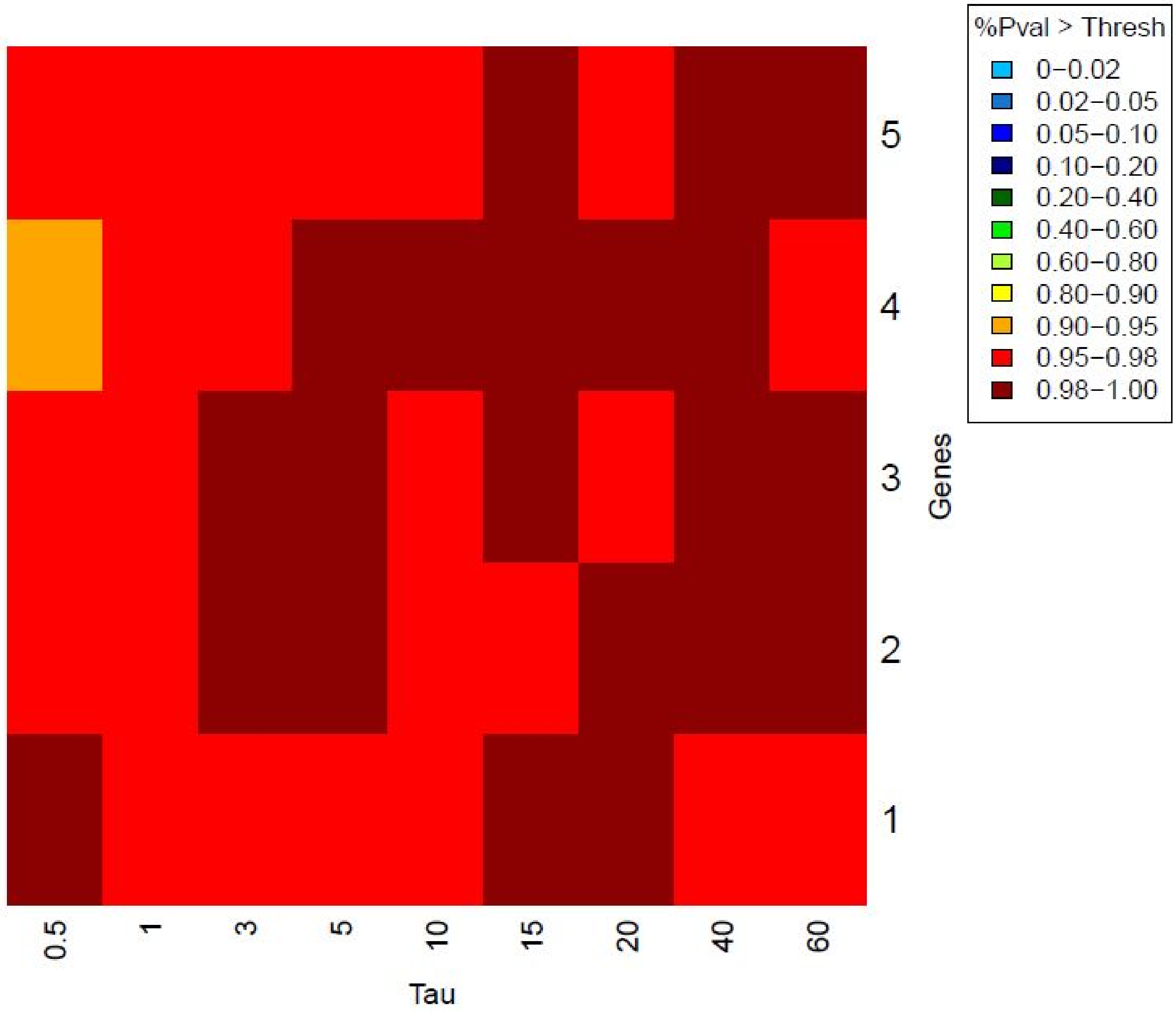

Supplemental Tables

Table S1. All the great ape copy number samples used in the study.

Species	IID
Gorilla	KB3781
Gorilla	KB10845
Gorilla	KB14216
Gorilla	KB15257
Gorilla	KB15813
Gorilla	KB3456
Gorilla	KB3512
Gorilla	KB4987
Gorilla	KB4988
Gorilla	KB4989
Gorilla	KB5712
Gorilla	KB6319
Gorilla	KB7026
Gorilla	KB7801
Chimpanzee	Bandit
Chimpanzee	BigDaddy
Chimpanzee	Conan
Chimpanzee	Moose
Chimpanzee	Rock
Chimpanzee	Budda
Chimpanzee	Cordova
Chimpanzee	Neptune
Chimpanzee	Zippy
Bornean orangutan	KB13383
Bornean orangutan	KB3042
Bornean orangutan	KB4204

Bornean orangutan	KB5418
Bornean orangutan	KB5419
Bornean orangutan	KB6109
Bornean orangutan	KB9002
Bonobo	KB1843
Bonobo	KB3841
Bonobo	KB4229
Bonobo	KB7032
Bonobo	KB7781
Bonobo	KB7782
Bonobo	KB7998
Sumatran orangutan	KB4650
Sumatran orangutan	KB4661
Sumatran orangutan	KB5390
Sumatran orangutan	KB5565
Sumatran orangutan	KB5883
Human	7
Human	8
Human	17
Human	23
Human	42
Human	46
Human	47
Human	48
Human	67
Human	72

Table S2. List of RNA-Seq samples.

**Species**	**NCBI SRA ID**	**Replicates**	**Sequencing Type**	**Tissue**
**Bornean orangutan**	SRR2176206, SRR2176207	2	Paired end	Testis
**Bornean orangutan**	In lab (3405): SRR10392514-SRR10392519	6	Paired End	Testis
**Sumatran orangutan**	SRR10393299-SRR10393304	6	Paired End	Testis
**Bornean orangutan**	SRR306798	1	Single End	Liver
**Gorilla**	SRR306810	1	Single End	Testis
**Gorilla**	SRR3053573, SRR10393358	2	Paired End	Testis
**Gorilla**	SRR306808	1	Single end	Liver
**Chimpanzee**	SRR306825	1	Single End	Testis
**Chimpanzee**	SRR2040590	1	PairedEnd	Testis
**Chimpanzee**	SRR2040591	1	Paired End	Testis
**Chimpanzee**	SRR306823	1	Single End	Liver
**Bonobo**	SRR306837	1	Single End	Testis
**Bonobo**	In lab (5013): SRR10392519-SRR10392521	3	Paired End	Testis
**Bonobo**	SRR306835	1	Single End	Liver
**Human**	SRR1090722	1	Paired End	Testis
**Human**	SRR306825	1	Paired End	Testis
**Human**	SRR817512	1	Paired End	Testis
**Human**	SRR1100440	1	Paired End	Testis
**Human**	SRR1071668	1	Paired End	Liver

Table S3. Mean values of ampliconic gene copy numbers across at least three ddPCR replicates.

Species	IID	*BPY2*	*CDY*	*DAZ*	*HSFY*	*PRY*	*RBMY*	*TSPY*	*VCY*	*XKRY*
B. orangutan	KB13383	16.01	49.64	12.04	5.56	10.29	4.03	43.56	NA	19.24
B. orangutan	KB3042	21.07	39.31	12.59	4.4	7.55	4.19	47.7	NA	14.86
B. orangutan	KB4204	12.77	30.15	9.73	4.36	6.77	2.83	29.08	NA	15.21
B. orangutan	KB5418	14.83	34.99	10.52	4.86	10.33	3.39	32.23	NA	17.17
B. orangutan	KB5419	13.89	30.04	9.69	4.71	8.46	2.96	30.44	NA	13.78
B. orangutan	KB6109	21.54	46.61	10.65	6.21	7.98	4.05	35.3	NA	19.81
B. orangutan	KB9002	13.28	30.38	9.81	5.58	8.79	2.67	28.62	NA	13.99
Bonobo	KB1843	1.12	3.09	2.19	NA	NA	31.52	51.86	NA	NA
Bonobo	KB3841	1.06	2.98	1.86	NA	NA	28.8	48.07	NA	NA
Bonobo	KB4229	2.15	3.25	2.13	NA	NA	28.32	44.64	NA	NA
Bonobo	KB7032	1.96	2.99	2.06	NA	NA	28.59	52.19	NA	NA
Bonobo	KB7781	1.06	3.09	2.03	NA	NA	29.57	48.41	NA	NA
Bonobo	KB7782	0.84	2.49	1.65	NA	NA	23.13	39.31	NA	NA
Bonobo	KB7998	0.98	2.93	1.87	NA	NA	22.53	38.09	NA	NA
Chimpanzee	Bandit	1.84	4.59	3.46	NA	NA	6.25	11.61	1.88	NA
Chimpanzee	Big Daddy	2.19	5.47	4.44	NA	NA	12.01	11.54	2.25	NA
Chimpanzee	Budda	2.2	5.52	4.33	NA	NA	9.11	15.85	2.09	NA
Chimpanzee	Conan	2.03	5.28	4.3	NA	NA	9.65	17.62	2.05	NA
Chimpanzee	Cordova	2.03	4.98	4.09	NA	NA	10.46	15.15	2.03	NA
Chimpanzee	Moose	2.05	5.25	4.09	NA	NA	11.1	25.45	2.07	NA
Chimpanzee	Neptune	2.3	5.5	4.31	NA	NA	10.86	23.67	2.13	NA
Chimpanzee	Rock	1.97	4.94	3.82	NA	NA	11	20.96	1.99	NA
Chimpanzee	Zippy	2.21	5.42	4.35	NA	NA	12.18	21.51	2.08	NA
Gorilla	KB10845	1.97	9.82	1.96	7.84	2.04	13.71	5.87	NA	2.01
Gorilla	KB14216	2	6.01	2.07	3.97	1.96	15.86	6	NA	1.98
Gorilla	KB15257	2.11	6.22	2.07	4.14	2.06	14.43	8.21	NA	2.05
Gorilla	KB15813	1.97	7.9	2.06	5.98	1.99	13.8	7.88	NA	2
Gorilla	KB3456	2.17	7.81	2.17	5.93	2.08	15.91	7.93	NA	1.99
Gorilla	KB3512	1.95	6.13	2.02	4.09	2.08	14.1	6	NA	2.03
Gorilla	KB3781	1.98	8.32	1.97	6.1	2.02	16.14	8.08	NA	1.98
Gorilla	KB4987	1.96	5.99	1.98	4.03	1.97	13.9	6.04	NA	1.97
Gorilla	KB4988	2.04	9.87	2.01	7.97	1.99	13.85	6	NA	1.96
Gorilla	KB4989	2.01	8.07	2.02	6.06	2.05	12.03	6.06	NA	2.06
Gorilla	KB5712	2.02	5.91	1.96	4.95	2.09	13.73	5.84	NA	1.95
Gorilla	KB6319	2.1	6.09	2	4.14	2.13	18.06	8.1	NA	2.01

Gorilla	KB7026	2.13	12.17	2.08	8.19	2.07	14.21	6.08	NA	2.03
Gorilla	KB7801	2.04	8.34	2.11	6.23	2.12	14.7	8.24	NA	2.09
Human	17	3.31	4.4	5.27	2.28	2.15	10.66	33.04	2.14	2.14
Human	23	2.76	3.47	4.51	1.58	2.49	4.95	35.29	2.12	1.55
Human	42	1.68	3.08	4.81	1.58	2	7.63	26.03	2.03	1.53
Human	46	3.67	4.63	4.49	2.28	2.03	6.82	36.69	2.09	2.2
Human	47	3.49	3.85	4.36	2.04	2.18	11.03	32.88	3.17	1.96
Human	48	2.15	2.87	2.78	2.09	2.78	9.32	32.24	2.09	2.01
Human	67	3.3	4.36	4.33	2.29	2.08	9.69	39.09	2.03	2.17
Human	7	3.22	4.26	4.31	2.05	2.12	10.41	33.03	2.38	1.95
Human	72	2.88	3.5	2.99	1.95	1.59	9.18	31.24	1.67	1.93
Human	8	3.34	4.54	4.37	2.27	2.21	10.71	35.72	2.38	2.21
S. orangutan	KB4650	13.38	35.75	13.84	5.98	10.69	2.8	31.96	NA	23.19
S. orangutan	KB4661	12.43	33.16	11.55	4.95	9.9	2.78	21.31	NA	20.49
S. orangutan	KB5390	13.64	37.72	12.39	6.47	10.17	2.98	22.83	NA	25.52
S. orangutan	KB5565	13.69	39.58	11.84	5.26	9.59	3.08	14.04	NA	21.06
S. orangutan	KB5883	25.33	34.3	12.97	5.49	10.2	2.79	29.55	NA	22.19

Table S4. Summary of ampliconic gene copy numbers across great apes.

The median, variance, and range of each individual gene family and all families together in each species studied.

Gene Families	Bonobo				Chimpanzee			
	Median	Variance	Min	Max	Median	Variance	Min	Max
BPY2	1.06	0.27	0.84	2.15	2.05	0.02	1.84	2.3
CDY	2.99	0.06	2.49	3.25	5.28	0.1	4.59	5.52
DAZ	2.03	0.04	1.65	2.19	4.3	0.1	3.46	4.44
HSFY	0	0	0	0	0	0	0	0
PRY	0	0	0	0	0	0	0	0
RBMY	28.59	11.3	22.53	31.52	10.86	3.27	6.25	12.18
TSPY	48.07	31.98	38.09	52.19	17.62	25.5	11.54	25.45
VCY	0	0	0	0	2.07	0.01	1.88	2.25
XKRY	0	0	0	0	0	0	0	0

Gene Families	Human				Gorilla			
	Median	Variance	Min	Max	Median	Variance	Min	Max
BPY2	3.26	0.4	1.68	3.67	2.01	0.01	1.95	2.17
CDY	4.06	0.4	2.87	4.63	7.86	3.57	5.91	12.17
DAZ	4.37	0.58	2.78	5.27	2.02	0	1.96	2.17
HSFY	2.07	0.07	1.58	2.29	5.95	2.34	3.97	8.19
PRY	2.13	0.1	1.59	2.78	2.06	0	1.96	2.13
RBMY	9.5	3.93	4.95	11.03	14.16	2.15	12.03	18.06
TSPY	33.04	12.54	26.03	39.09	6.07	1.16	5.84	8.24
VCY	2.11	0.15	1.67	3.17	0	0	0	0
XKRY	1.98	0.06	1.53	2.21	2	0	1.95	2.09

Gene Families	B. Orangutan				S. Orangutan			
	**Median**	**Variance**	**Min**	**Max**	**Median**	**Variance**	**Min**	**Max**
BPY2	14.83	13.3	12.77	21.54	13.64	29.29	12.43	25.33
CDY	34.99	66.68	30.04	49.64	35.75	6.69	33.16	39.58
DAZ	10.52	1.36	9.69	12.59	12.39	0.85	11.55	13.84
HSFY	4.86	0.49	4.36	6.21	5.49	0.36	4.95	6.47
PRY	8.46	1.79	6.77	10.33	10.17	0.16	9.59	10.69
RBMY	3.39	0.41	2.67	4.19	2.8	0.02	2.78	3.08
TSPY	32.23	56.38	28.62	47.7	22.83	50.47	14.04	31.96
VCY	0	0	0	0	0	0	0	0
XKRY	15.21	6.11	13.78	19.81	22.19	3.95	20.49	25.52

Table S5. *P*-values from permutation tests for copy number differences between Sumatran and Bornean orangutans.

Given two species, we tested whether the difference in copy number between the species is significant. We compared the true difference in mean copy number between the species to the difference in mean of one million random permutations (randomly rearranged the species assignment of the two species). The *p*-value represents the fraction of permuted mean differences that are larger than the one we observed in our actual data. The *p*-values that pass a Bonferroni corrected cutoff for eight tests (0.05/8 = 0.00625) are highlighted in bold.

**Gene family**	***p*-value**
BPY2	0.863
CDY	0.747
DAZ	0.02
HSFY	0.195
PRY	0.042
RBMY	0.094
TSPY	0.028
XKRY	**0.001**

Table S6. The branch-level *p*-values showing the presence of significant shift in copy number when compared to its immediate ancestor in the great ape phylogenetic tree. The columns represent the branches in the great ape phylogenetic tree and the rows represent gene families with significant expansions or contractions in one or more branches as predicted by CAFE.

**Gene family**	**Bonobo**	**Chimpanzee**	**(Bonobo, Chimp)**	**Human**	**(Bonobo, Chimp, Human)**	**Gorilla**	**(Bonobo, Chimp, Human, Gorilla)**	**Orangutans**	**Bornean orangutan**	**Sumatran orangutan**
CDY	0.344	0.158	0.577	0.644	0.246	0.476	0.042	**1.86×10^{-3}**	0.814	0.298
RBMY	**2.02×10^{-7}**	**9.61×10^{-4}**	0.105	0.312	0.522	0.4	0.292	0.195	0.557	0.557
TSPY	**1.39×10^{-9}**	**3.89×10^{-7}**	0.324	0.115	0.032	**5.07×10^{-5}**	0.802	0.43	**2.68×10^{-4}**	**1.01×10^{-3}**
XKRY	0.5	0.5	0.026	0.79	0.716	0.843	0.135	**4.31×10^{-3}**	**2.86×10^{-3}**	**6.46×10^{-4}**

Table S7. Summary of CAFE results with five individuals per species added as star phylogeny.

Each number in the table represents the number of times out of the 100 simulations in which CAFE estimated a significant shift in copy number ($p<0.005$). For each simulation, ampliconic gene copy numbers from five random individuals per species were used in CAFE analysis to capture the copy number variation within each species. Columns represent the branches of the phylogenetic tree. Rows represent ampliconic gene families. The numbers in bold are the branches with observed significant shifts in copy number when median copy number per species was used in CAFE analysis (Table S6).

Gene family	Bonobo	Chimp	(Bonobo, Chimp)	Human	(Bonobo, Chimp, Human)	Gorilla	(Bonobo, Chimp, Human, Gorilla)	Orangutans	Bornean orangutan	Sumatran orangutan
BPY2	1	0	1	0	0	0	15	15	4	0
CDY	15	0	0	3	9	0	15	**100**	5	2
DAZ	15	0	0	0	0	7	5	11	0	9
HSFY	0	0	13	0	14	1	0	0	0	3
PRY	0	0	15	0	0	0	6	12	0	12
RBMY	**100**	**83**	0	2	0	6	3	14	0	22
TSPY	**99**	**99**	0	15	15	**84**	15	15	**57**	**39**
VCY	4	11	0	4	0	1	0	0	0	0
XKRY	0	0	8	0	1	0	15	**55**	**12**	**68**

Table S8. Gene expression values for Y ampliconic gene families across great apes.

Numbers represent read counts after normalization. The read counts for Y ampliconic gene families missing in great apes are represented as NA.

	Bonobo		Chimpanzee			Human			Gorilla		B. orangutan		S. Orangutan
**Gene family**	**SRR10392521 (this study)**	**SRR306837**	**SRR2040590**	**SRR2040591**	**SRR306825**	**SRR1100440**	**SRR817512**	**SRR1102852**	**SRR3053573**	**SRR306810**	**SRR10392517 (this study)**	**SRR2176206**	**SRR10393300**
BPY2	25.79	79.63	30.49	65.6	58.19	39.66	78.9	62.82	0	4.42	40.17	4.71	36.65
CDY	115.5	97.32	201.21	388.88	793.28	77.89	258.37	157.29	52.09	85.44	107.11	53.64	2040.28
DAZ	274.72	183.58	646.94	420.81	274.12	611.46	493.15	630.82	277.79	251.9	1071.1	644.59	134.39
HSFY	NA	NA	NA	NA	NA	219.13	436.96	588.14	1820.86	290.2	2155.58	558.95	5558.86
PRY	NA	NA	NA	NA	NA	13.98	26.42	38.36	0	0	0	0.94	0
RBMY	590.93	453.42	934.47	453.48	356.82	492.76	519.57	575.19	403.67	474.34	66.94	46.11	0
TSPY	913.87	1431.04	530.45	1376.91	722.83	2329.14	1620.16	2013.06	883.3	212.13	1332.18	604.12	598.65
VCY	NA	NA	386.37	288.1	73.51	883.38	862.83	1179.4	NA	NA	NA	NA	NA
XKRY	NA	NA	NA	NA	NA	0.57	0.7	0.96	0	0	0	0	0

Table S9. EVE-model-based likelihood ratios and *p*-values showing no significant shift in gene expression of shared ampliconic gene families across great apes.

Gene family	Likelihood ratio	*p*-value
BPY2	0.1064296	0.744
CDY	0.2014877	0.654
DAZ	0.2407138	0.623
RBMY	0.01832644	0.892
TSPY	0.02637181	0.871

Supplemental Figures

Figure S1. Transcriptome assembly pipeline.

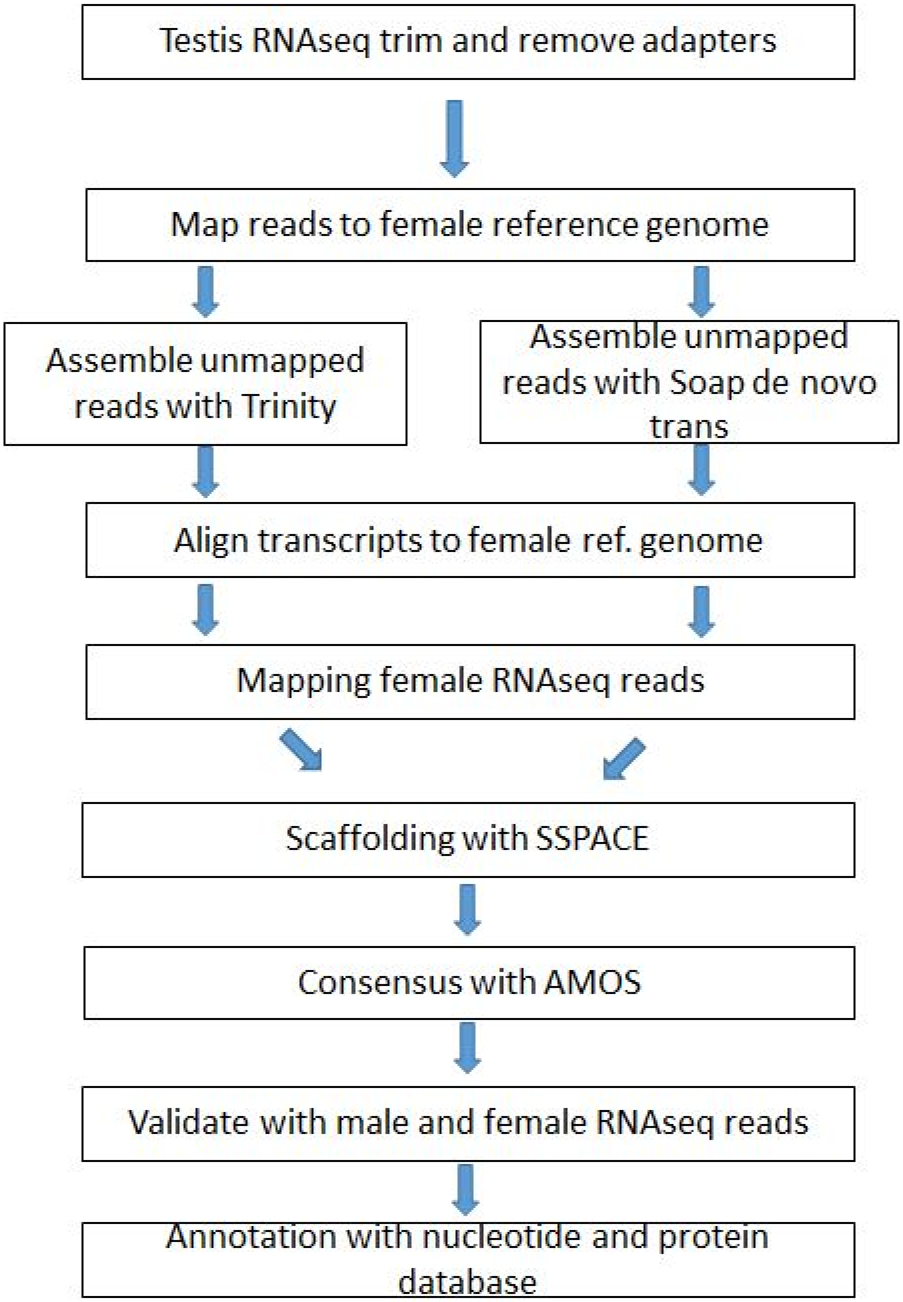

Figure S2. Heatmap depicting the Euclidean distances of gene expression values between pairs of sampled individuals.

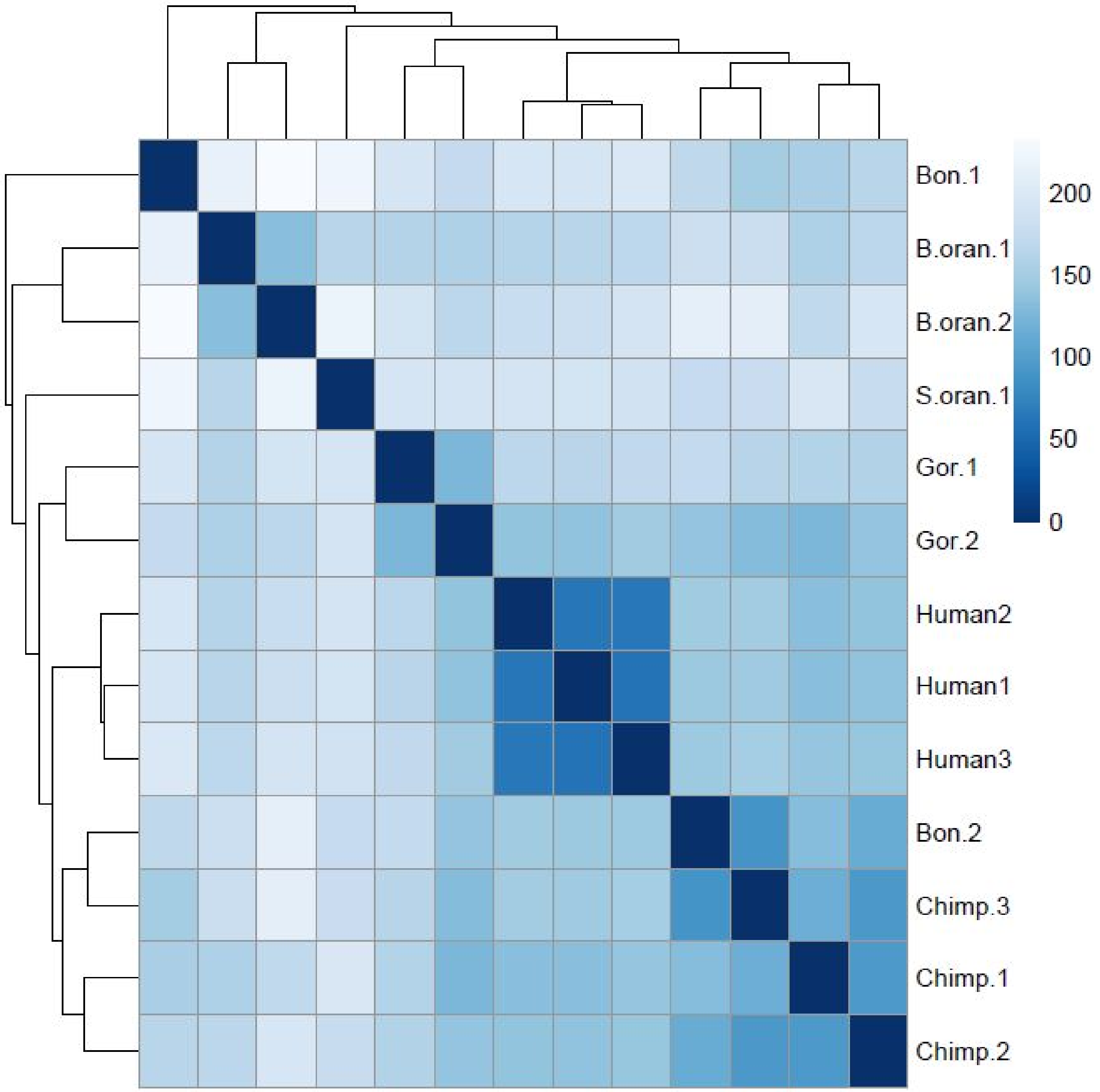

Figure S3. Principal components analysis of great ape gene expression values (RLD-normalized).

The gene expression values were normalized using regularized log transformation *rlog()* function in DESeq2 (Love et al. 2014).

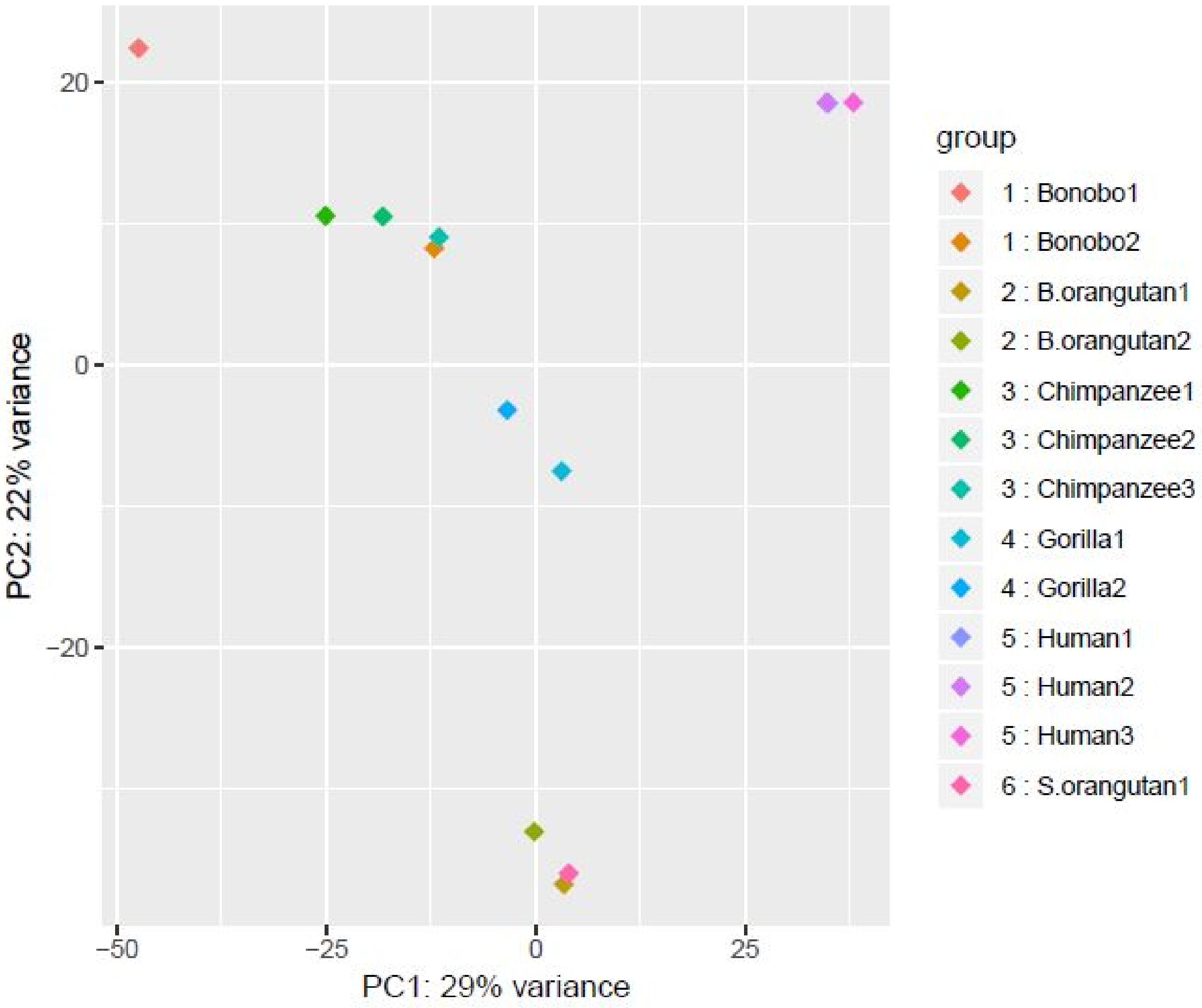

Figure S4. Principal components analysis of great ape gene expression values (VST-normalized).

Gene expression values were normalized using Variance Stabilizing Transformation *varianceStabilizingTransformation()* function in DESeq2 (Love et al. 2014).

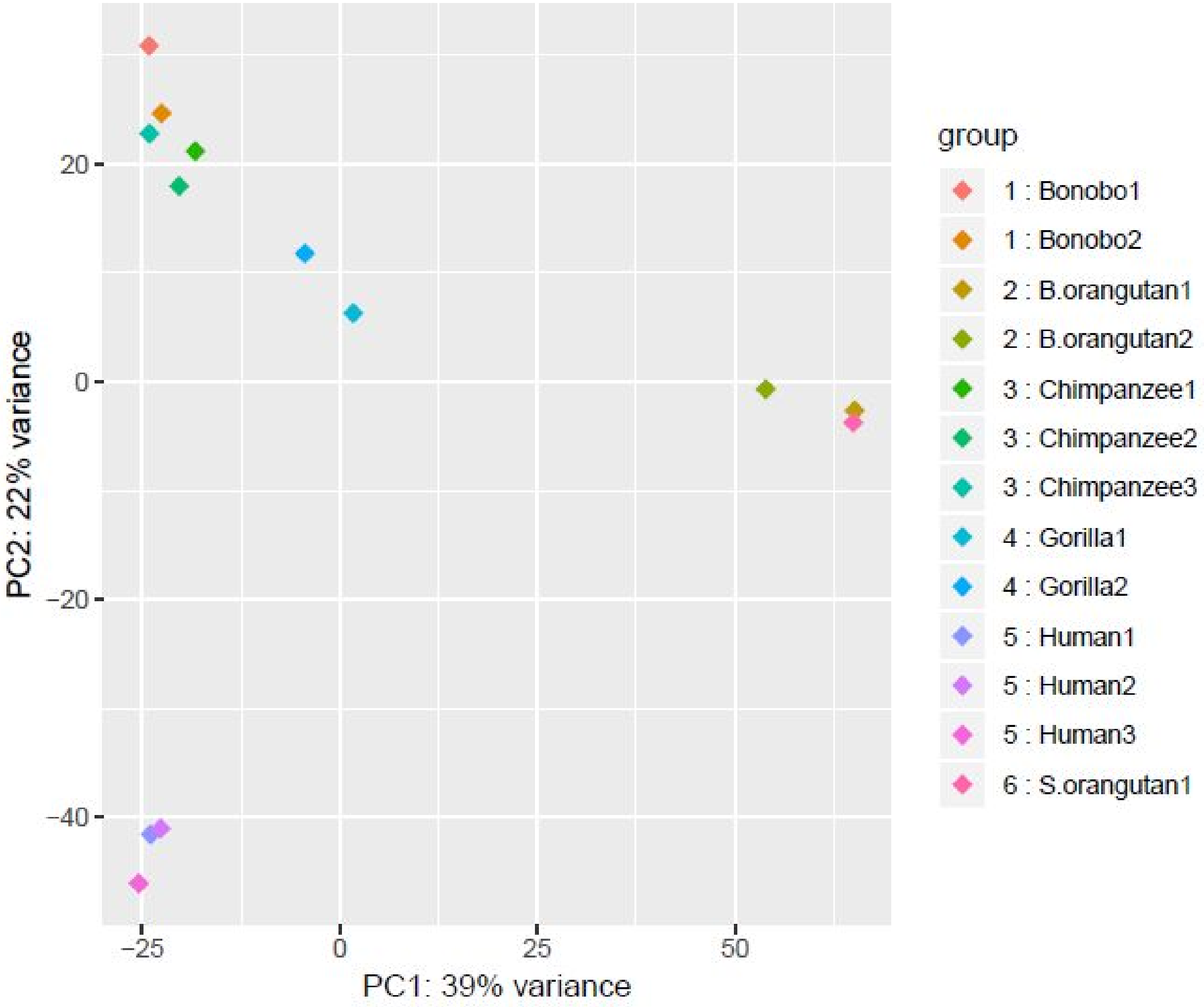

Figure S5. Principal components analysis of the overall copy number of Y ampliconic gene families across great apes.

Proportion of total variance explained by the first five principal components (PCs) is on the Y-axis. The first, second, third, fourth, and fifth PCs explained 68.7%, 22.8%, 6.5%, 1.2%, and 0.8% of the variation, respectively.

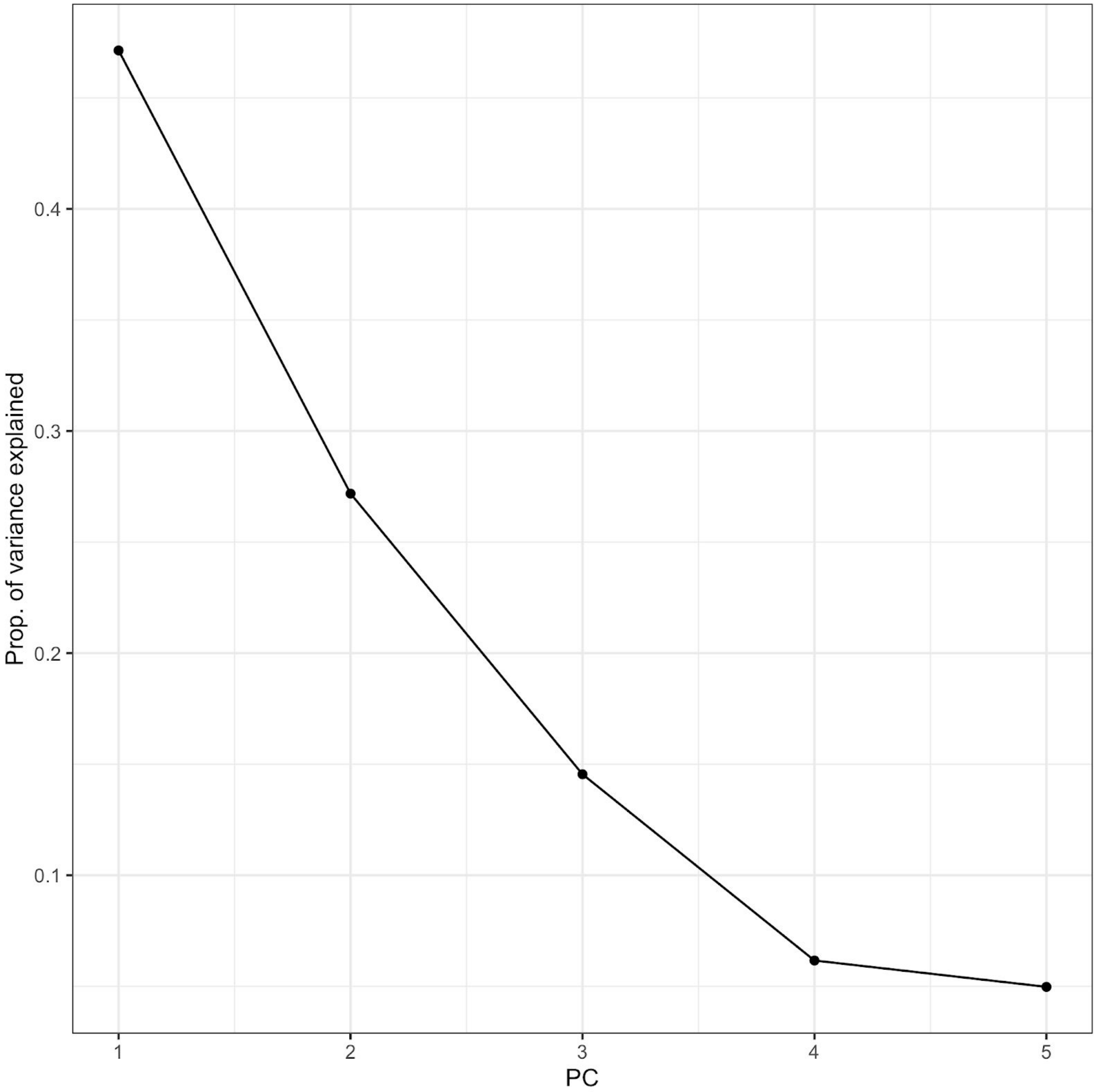

Figure S6. Across species, gene families with higher copy number have higher variance. In each of the scatterplots the X-axis represents natural log of median copy number and the Y-axis represents natural log of variance in copy number. The Spearman correlations were calculated using the *cor.test()* function in R and the *p*-values are in parentheses. The black line represents the linear function fitted to the given data points. The dots are color-coded to represent the six species, with missing dots indicating that the corresponding gene families are either lost or pseudogenized in that species.

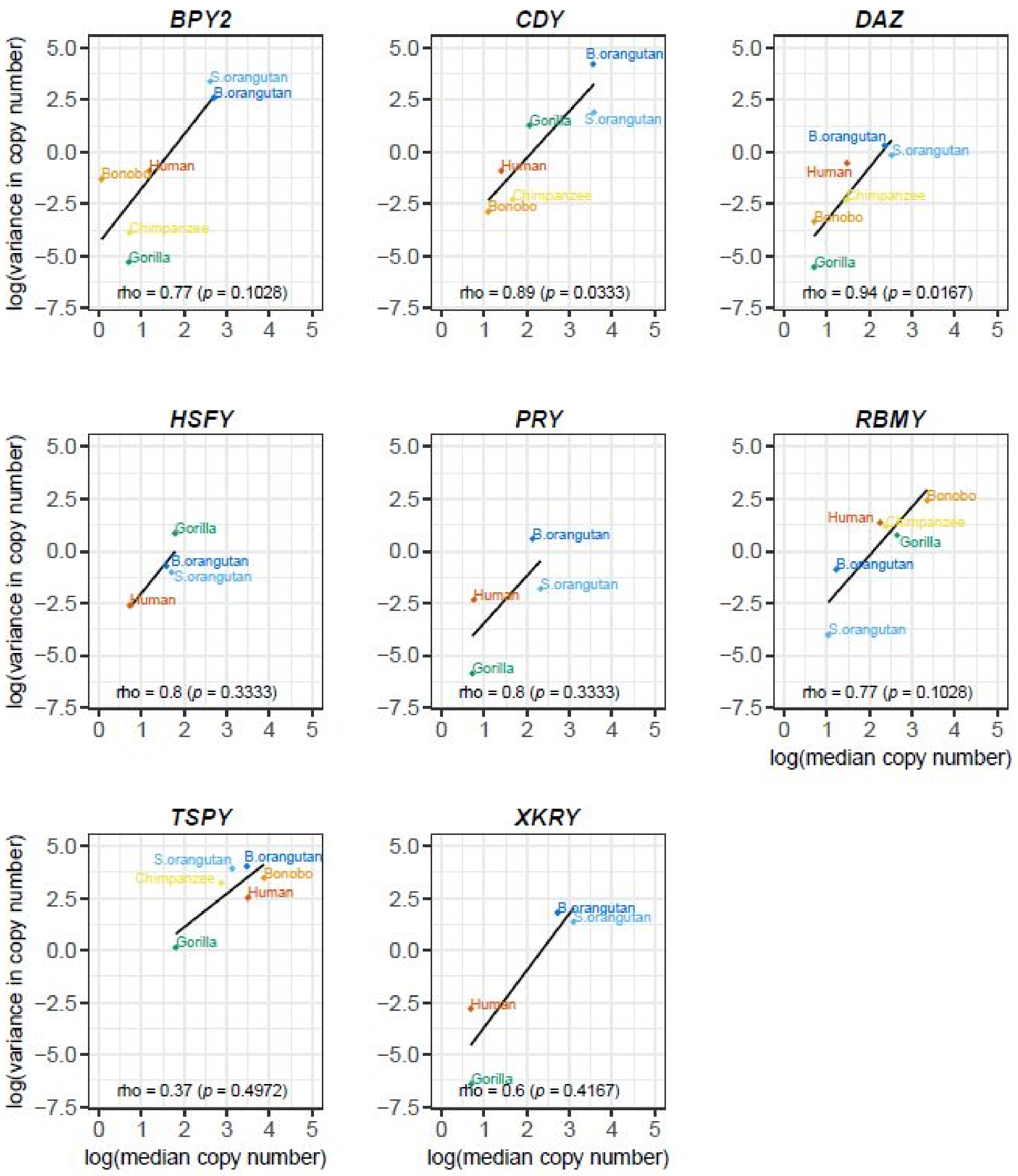

Figure S7. Heatmap depicting the Euclidean distances of gene expression values (using assembled species-specific transcripts as a reference) between pairs of sampled individuals.

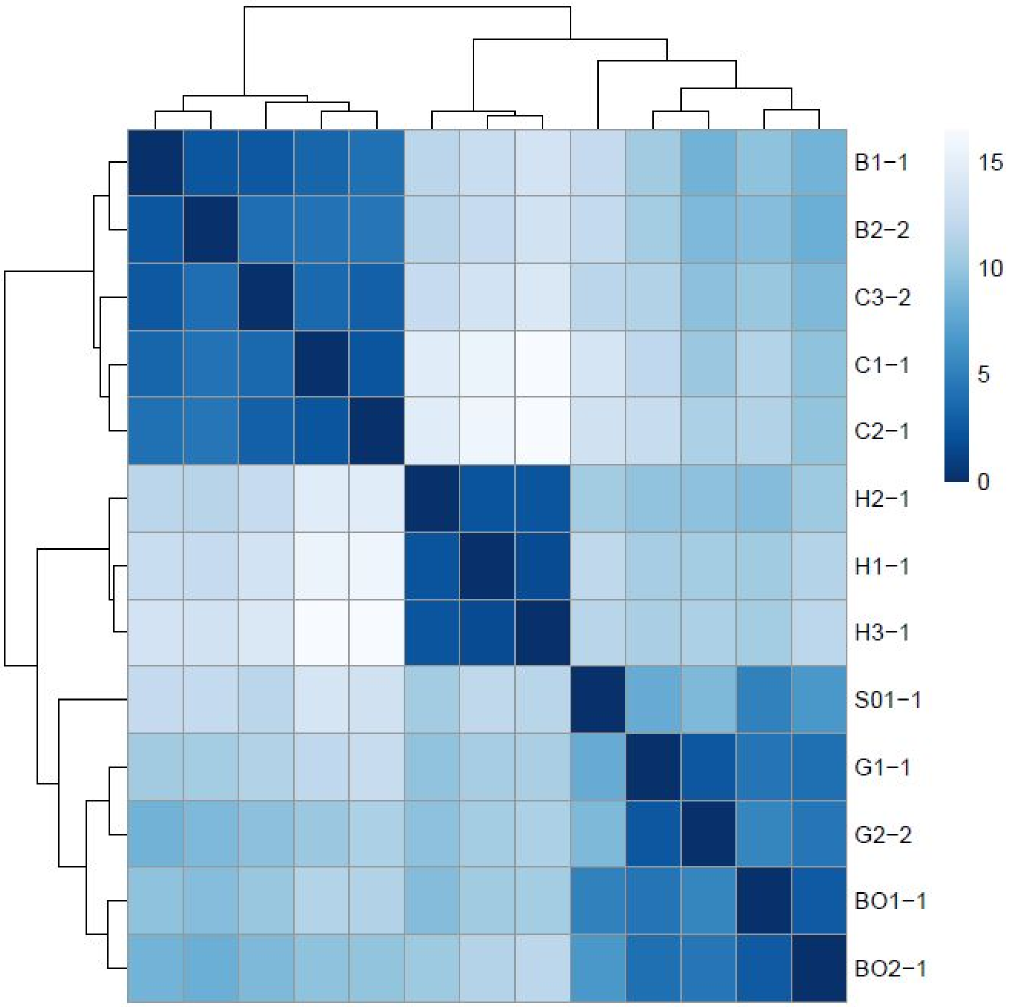

Figure S8. Summary of gene expression levels across great apes (using assembled species-specific transcripts as a reference).

In the dot plot below, the X-axis represents nine ampliconic gene families and the Y-axis represents their expression levels. The plot represents testis-specific expression of 12 great ape samples. Each dot within a gene family represents expression levels of an individual and the color of the dot denotes the species it belongs to. Missing dots represent gene families that are considered missing or pseudogenized, and their expression levels are excluded from the gene expression analysis.

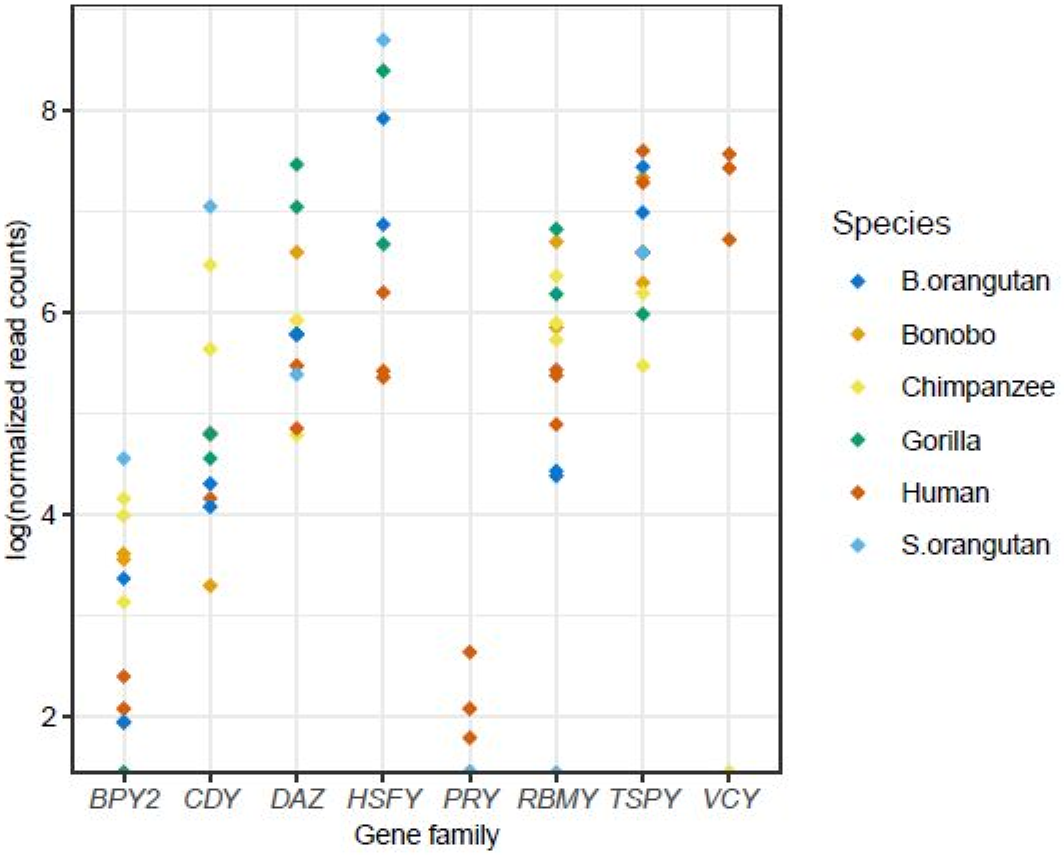

Figure S9. Relationship between copy number and gene expression (using assembled species-specific transcripts as a reference) of Y ampliconic gene families in great ape species.

The five scatter plots represent the relationship between expression and copy number for each of the five species, the name of the species is present at the top of each plot. In each of the scatter plots the X-axis represents natural logarithm of median copy number and the Y-axis represents natural logarithm of median gene expression. The Spearman correlations were calculated using the *cor.test()* function in R and the *p*-values are in parentheses. The black line is the linear function fitted to the given data points. The dots are color-coded to represent the nine gene families, with missing dots corresponding to the gene families that are pseudogenized, deleted, or not expressed, in that species.

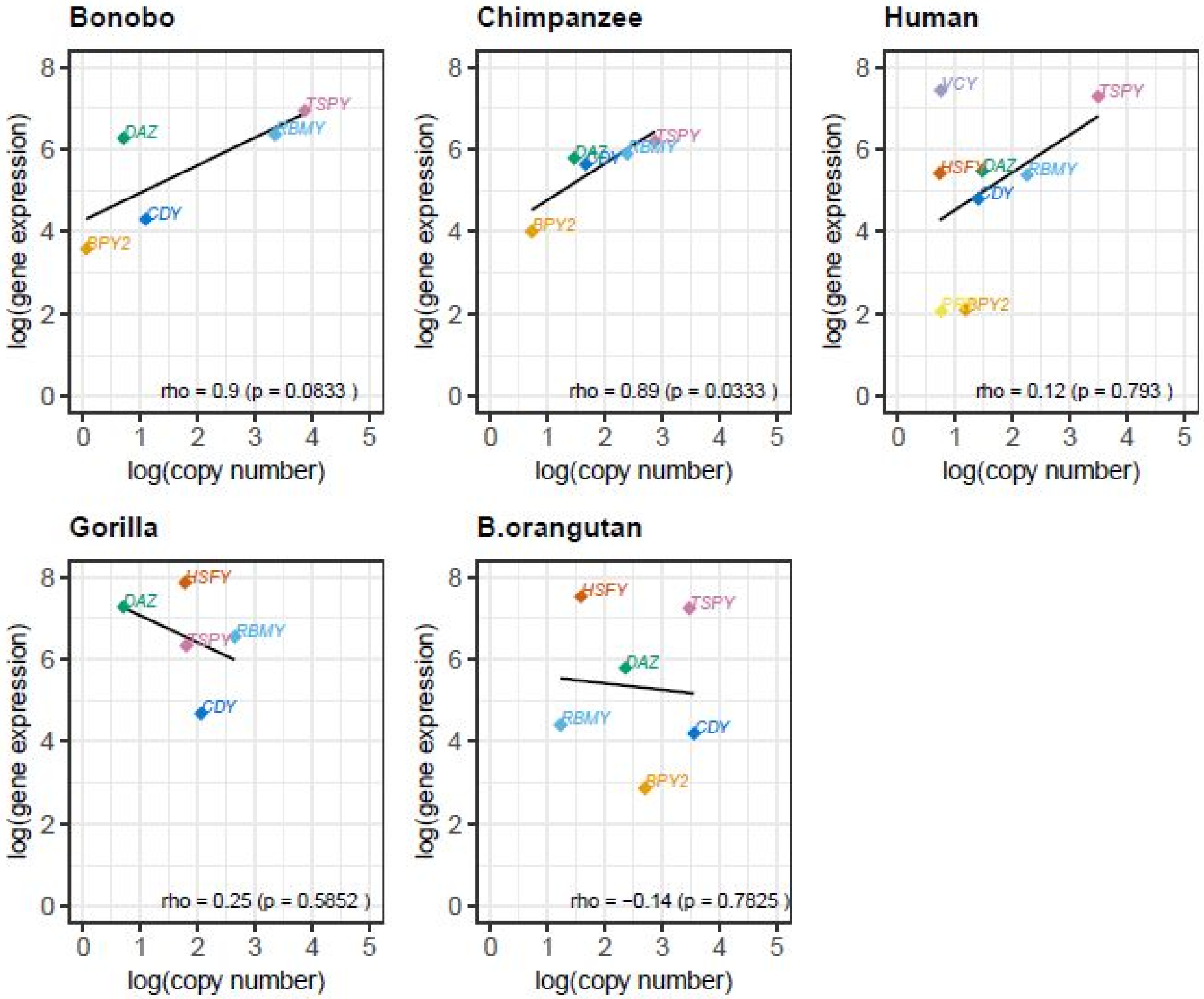

Figure S10. Relationship between copy number and gene expression (using assembled species-specific transcripts as a reference) across species.

In each of the scatter plots the X-axis represents natural logarithm of median copy number and the Y-axis represents natural logarithm of median gene expression. The Spearman correlations were calculated using the *cor.test()* function in R and the *p*-values are in parentheses. The black line represents the linear function fitted to the given data points. The dots are color-coded to represent the five species. The five scatter plots represent the relationship between expression and copy number for each of the five gene families, with the name of the gene family present at the top of each plot. Only the gene families that are present in all species are shown here.

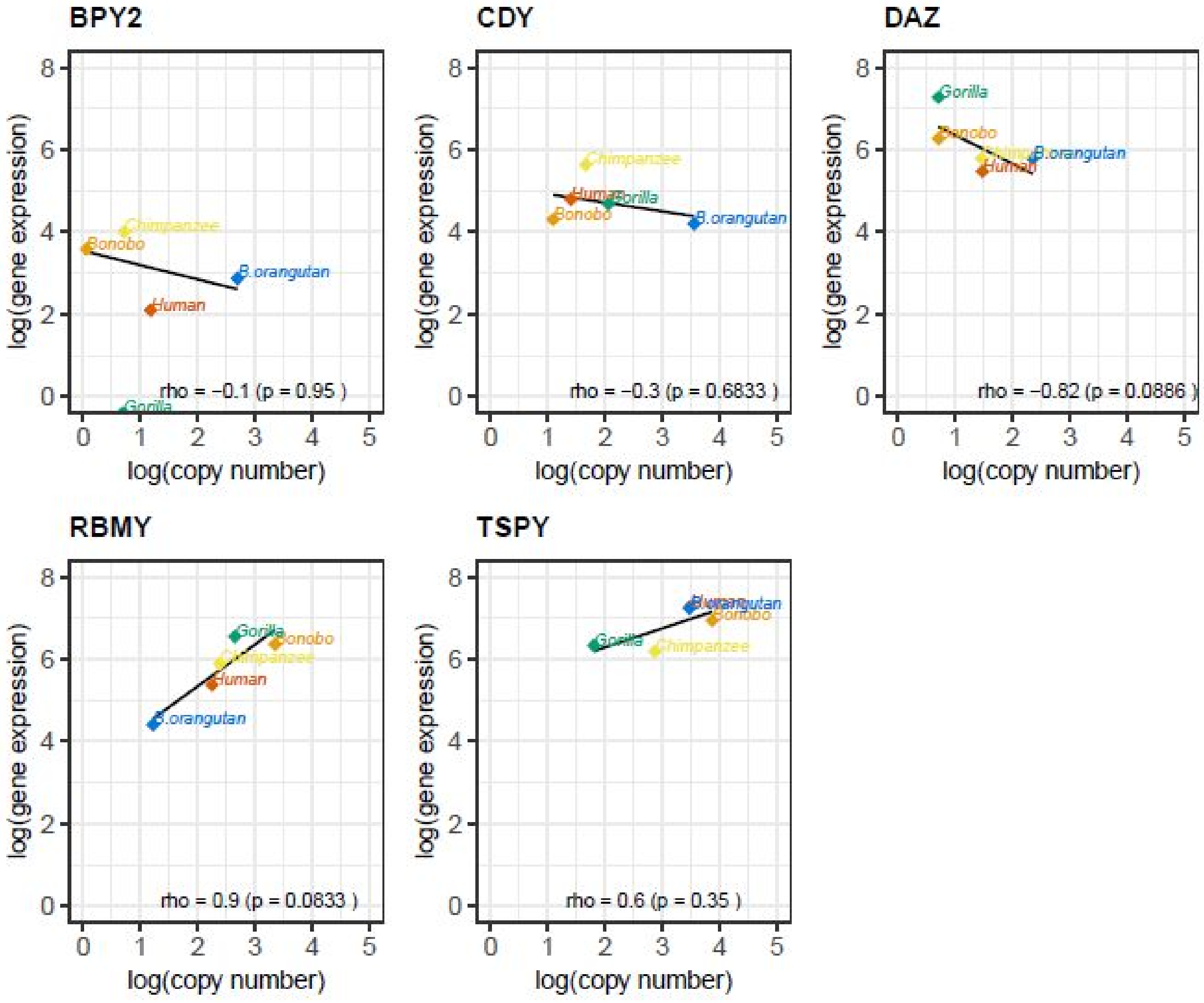

References

Ardlie KG et al. 2015. The Genotype-Tissue Expression (GTEx) pilot analysis: Multitissue gene regulation in humans. Science. 348:648–660.

Hahn MW, Demuth JP, Han S-G. 2007. Accelerated rate of gene gain and loss in primates. Genetics. 177:1941–1949.

Hallast P et al. 2016. Great ape Y Chromosome and mitochondrial DNA phylogenies reflect subspecies structure and patterns of mating and dispersal. Genome Res. 26:427–439.

Han MV, Thomas GWC, Lugo-Martinez J, Hahn MW. 2013. Estimating gene gain and loss rates in the presence of error in genome assembly and annotation using CAFE 3. Mol. Biol. Evol. 30:1987–1997.

Locke DP et al. 2011. Comparative and demographic analysis of orang-utan genomes. Nature. 469:529–533.

Love MI, Huber W, Anders S. 2014. Moderated estimation of fold change and dispersion for RNA-seq data with DESeq2. Genome Biol. 15:550.

Rohlfs R, Nielsen R. 2014. Phylogenetic ANOVA: The Expression Variance and Evolution (EVE) model for quantitative trait evolution. doi: 10.1101/004374.

Rohlfs RV, Harrigan P, Nielsen R. 2014. Modeling gene expression evolution with an extended Ornstein-Uhlenbeck process accounting for within-species variation. Mol. Biol. Evol. 31:201–211.

Rohlfs RV, Nielsen R. 2015. Phylogenetic ANOVA: The expression variance and evolution model for quantitative trait evolution. Syst. Biol. 64:695–708.
